# Protocol for production and purification of SARS-CoV-2 3CL^pro^

**DOI:** 10.1016/j.xpro.2023.102326

**Published:** 2023-05-05

**Authors:** Luca Mazzei, Rebecca Greene-Cramer, Khushboo Bafna, Aleksandar Jovanovic, Anna De Falco, Thomas B. Acton, Catherine Ann Royer, Stefano Ciurli, Gaetano T. Montelione

**Affiliations:** 1 Department of Pharmacy and Biotechnology, University of Bologna, 40127 Bologna, Italy; 2 Center for Biotechnology and Interdisciplinary Sciences, Rensselaer Polytechnic Institute, Troy, NY 12180, USA; 3 Department of Chemistry and Chemical Biology, Rensselaer Polytechnic Institute, Troy, NY 12180, USA; 4 Department of Biology, Rensselaer Polytechnic Institute, Troy, NY 12180, USA

**Keywords:** Microbiology, Protein Biochemistry, Structural Biology

## Abstract

3CL^pro^ protease from SARS-CoV-2 is a primary target for COVID-19 antiviral drug development. Here, we present a protocol for 3CL^pro^ production in *Escherichia coli*. We describe steps to purify 3CL^pro^, expressed as a fusion with the *Saccharomyces cerevisiae* SUMO protein, with yields up to 120 mg L^−1^ following cleavage. The protocol also provides isotope-enriched samples suitable for nuclear magnetic resonance (NMR) studies. We also present methods to characterize 3CL^pro^ by mass spectrometry, X-ray crystallography, heteronuclear NMR, and a Förster-resonance-energy-transfer-based enzyme assay.

For complete details on the use and execution of this protocol, please refer to Bafna et al*.*^1^

## Highlights

• Production and purification of SARS-CoV-2 3CL^pro^with native N- and C-termini
• 3CL^pro^inhibition assay and description for data processing/analysis
• Structural characterization by X-ray crystallography and NMR spectroscopy
• Detailed troubleshooting and alternative approaches

## Abstract (editor highlights)

Publisher’s note: Undertaking any experimental protocol requires adherence to local institutional guidelines for laboratory safety and ethics.

## Graphical abstract

**Figure:**
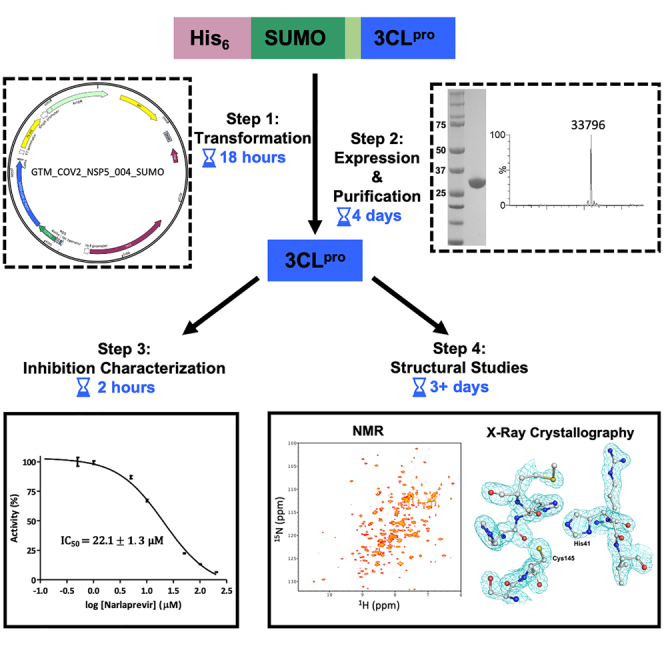

## Before you begin

Severe Acute Respiratory Syndrome CoronaVirus-2 3-Chymotrypsin-Like protease (SARS-CoV-2 3CL^pro^), also referred to as SARS-CoV-2 Main protease (M^pro^) or Non-structural Protein 5 (NSP5), is an essential viral cysteine protease required to hydrolyze the SARS CoV-2 1ab polyprotein at several sites during viral replication.^2^^,^^3^^,^^4^ It is recognized as a target for antiviral drug development. Structural and biochemical characterization of 3CL^pro^ inhibition to identify potential candidates for SARS-CoV-2 treatment and drug development rely on the production of large quantities of native and active 3CL^pro^. Protein – ligand binding at the molecular level, as well as the potency of enzyme inhibition, can be investigated using various techniques, such as heteronuclear NMR, biomolecular crystallography, and biochemical and kinetic assays.

In this work, we present a detailed and integrated protocol for 3CL^pro^ production, purification, and structural characterization, as well as for biochemical investigation of enzyme inhibition. High yields of native and active 3CL^pro^ (30–120 mg per liter of broth, depending on the type of broth used for fermentation) are routinely obtained using *Escherichia coli* cells. The protein is initially expressed as a fusion with an N-terminal His-SUMO tag (His_6_-SUMO-3CL^pro^), then purified to produce the native target upon tag hydrolysis by SUMO protease (for which a production and purification protocol is also described). Protocols to produce isotopically enriched (*e.g.*,^15^N,^13^C,^2^H-enriched) 3CL^pro^ samples, to structurally characterize the protein using heteronuclear NMR, as well as to crystalize 3CL^pro^ and determine the X-ray crystal structure at a resolution better than 2 Å, are also presented. These protocols provide a useful starting point for structural investigations of protein – inhibitor interactions. A detailed FRET-based enzyme assay for estimating IC_50_ values of 3CL^pro^ inhibitors is also reported.

SARS-CoV-2 3CL^pro^ is a high priority target for drug development and biophysical studies, and its production has been described previously as a component of other STAR Protocols.^5^^,^^6^ This work aims to provide, in a single and integrated set of protocols, a complete toolbox to produce large amounts of native and active SARS-CoV-2 3CL^pro^ and characterize the binding of drug candidates to this crucial viral target.

### Expression plasmids

#### His_6_-SUMO-3CL^pro^ expression plasmid

**Figure 1:**
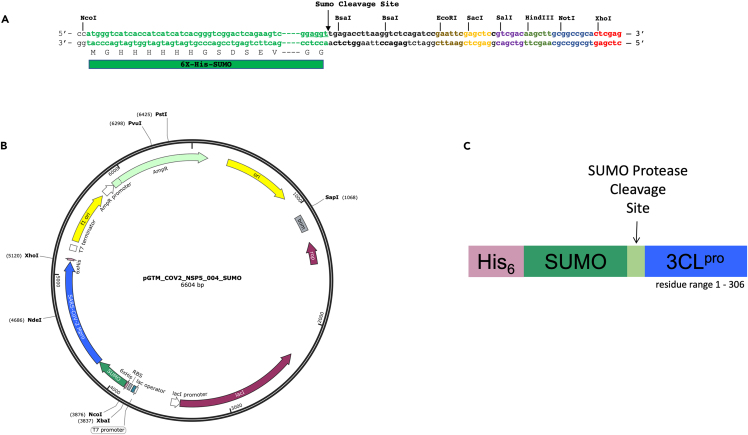
pGTM_CoV2_NSP5_004_SUMO construct design (A) Multiple cloning site of the original vector pET15_SUMO2_NESG showing BsaI restriction sites used for cloning the CDS of 3CL^pro^. Maps are generated using SnapGene® software (from Insightful Science; available at snapgene.com). The synthetic gene for 3CL^pro^ was obtained from Genscript, Inc. with standard Genscript codon optimization for *E. coli* expression. (B) Final expression vector pGTM_CoV2_NSP5_004_SUMO. (C) Schematic representation of the expressed His_6_-SUMO-3CL^pro^.

Plasmid pGTM_CoV2_NSP5_004_SUMO (Figure 1) was obtained by cloning the synthetic coding sequence (CDS) for SARS-CoV-2 3CL^pro^ (NSP5) into the pET15_SUMO2_NESG expression vector,^7^ which contains the CDS for an N-terminal hexa-His tag upstream to a SUMO tag and SUMO protease cleavage site. The plasmid has a pMB1 origin of replication and carries the ampicillin resistance gene. Plasmid pGTM_CoV2_NSP5_004_SUMO is available from AddGene (AddGene ID: 190062); the expressed protein construct will be called hereafter His_6_-SUMO-3CL^pro^. The related plasmid pGTM_CoV2_NSP5_C145A_001_SUMO, with active-site mutation Cys145Ala, is also available from AddGene (AddGene ID: 192349).

#### His_8_-MBP-SUMO^pro^ expression plasmid

**Figure 2:**
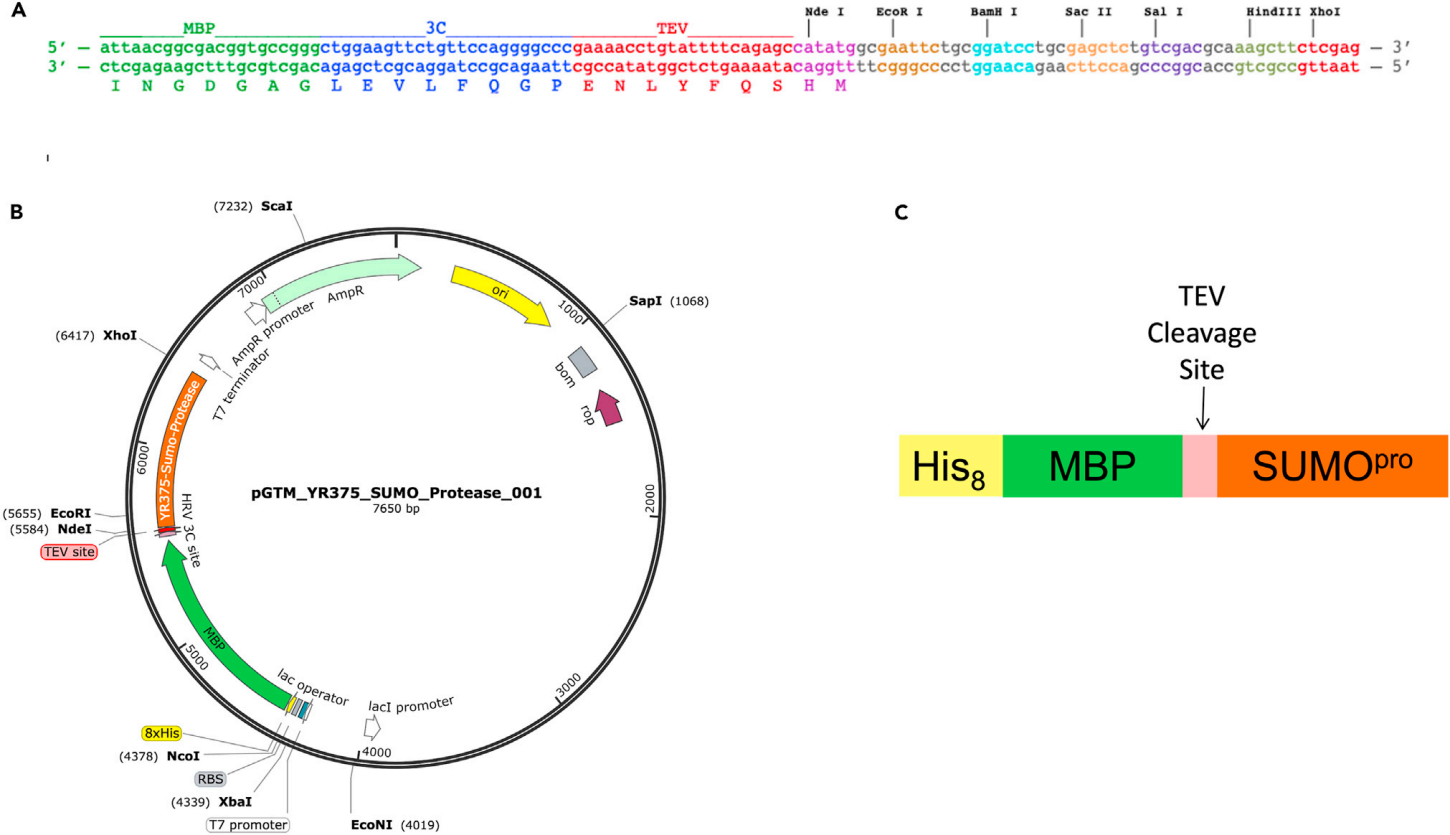
His_8_-MBP-SUMO^pro^ construct design (A) Multiple cloning site of the original vector pET15_8His_MBP_TEV_NESG. Maps are generated using SnapGene® software (from Insightful Science; available at snapgene.com). The synthetic gene for yeast SUMO^pro^ was obtained from Genscript, Inc. with standard Genscript codon optimization for *E. coli* expression. (B) Final expression vector pGTM_YR375 _SUMO_Protease_001. (C) Schematic representation of the expressed His_8_-MBP-SUMO^pro^ construct.

Plasmid pGTM_YR375_SUMO_Protease_001 (Figure 2) was obtained by cloning the CDS for residues 347–621 of the Ubiquitin-like-specific protease 1 from *Saccharomyces cerevisiae* [Uniprot ID: Q02724 (ULP1_YEAST)] into the pET15_8His_MbpTEV_NESG expression vector,^7^ which contains the CDS for an N-terminal octa-His tag upstream to Maltose Binding Protein Solubility Enhancing Tag (MBP tag) and TEV protease cleavage site. This plasmid has a pMB1 origin of replication and carries the ampicillin resistance gene. Plasmid pGTM_YR375_SUMO_Protease_001 is available from AddGene (AddGene ID: 190063) and the expressed protein construct will be called hereafter His_8_-MBP-SUMO^pro^.

### Preparation of reagent stock solutions

**Timing: 1 h**

1. Prepare 50 mg mL^−1^ ampicillin stock prepared in H_2_O (or ^2^H_2_O).
  a. Weigh 2.5 g (or 0.050 g) of ampicillin sodium salt (Sigma Aldrich).
  b. Dissolve it in a final volume of 50 mL (or 1.0 mL) using 50 % v/v ethanol solution (or ^2^H_2_O).
  c. Sterilize using 0.22 μm syringe filters and store at −20 °C.
2. Prepare 1 M isopropyl-β-d-thiogalactopyranoside (IPTG) in H_2_O (or ^2^H_2_O).
  a. Weigh 2.38 g (or 0.238 g) of IPTG (molar mass 238.30 g mol^−1^) (Sigma Aldrich).
  b. Dissolve it in a final volume of 10 mL (or 1.0 mL) using MilliQ water (or ^2^H_2_O).
  c. Sterilize using 0.22 μm syringe filters and store at −20 °C as 1 mL aliquots.
3. Prepare 1 M magnesium chloride (MgCl_2_) in H_2_O (or ^2^H_2_O).
  a. Weigh 95.2 g (or 0.30 g) of MgCl_2_ (molar mass 95.21 g mol^−1^) (Sigma Aldrich).
  b. Dissolve it in a final volume of 1 L (or 3.0 mL) using MilliQ water (or ^2^H_2_O).
  c. Filter the solution using 0.45 μm syringe filters and store at room temperature (20–25 °C).
4. Prepare 40 mg mL^−1^ deoxyribonuclease I from bovine pancreas (DNase I).
  a. Weigh 40.0 mg of Dnase I (Sigma Aldrich).
  b. Dissolve it in a final volume of 1 mL using MilliQ water.
  c. Store at −20 °C as 30 μL aliquots.
5. Prepare 1 M dithiothreitol (DTT).
  a. Weigh 1.54 g of DTT (molar mass 154.25 g mol^−1^) (Sigma Aldrich).
  b. Dissolve it in a final volume of 10 mL using MilliQ water.
  c. Store at −20 °C as 1 mL aliquots.
6. Prepare 0.1 M Tris(2-carboxyethyl)phosphine (TCEP).
  a. Weigh 286.7 mg of TCEP-HCl (molar mass 286.65 g mol^−1^) (Sigma Aldrich).
  b. Dissolve it in a final volume of 10 mL using MilliQ water.
  c. Store at −20 °C as 1 mL aliquots.
7. Prepare 0.1 M NiSO_4_.
  a. Weigh 1.55 g of anhydrous NiSO_4_ (molar mass 154.76 g mol^−1^) (Sigma Aldrich).
  b. Dissolve it in a final volume of 100 mL using MilliQ water.
  c. Filter the solution using 0.45 μm syringe filters and store at room temperature.

For production using MJ9 medium, also prepare Trace Element Stock in H_2_O or ^2^H_2_O, as appropriate:

8. Prepare 60 mL of trace elements stock.^8^
  a. Weigh 1.626 g of FeCl_3_ × 6H_2_O (molar mass 270.30 g mol^−1^) (Sigma Aldrich).
  b. Weigh 0.24 g of ZnSO_4_ × 7H_2_O (molar mass 287.56 g mol^−1^) (Sigma Aldrich).
  c. Weigh 0.42 g of CoCl_2_ × 6H_2_O (molar mass 237.93 g mol^−1^) (Sigma Aldrich).
  d. Weigh 0.42 g of Na_2_MoO_4_ × 2H_2_O (molar mass 241.95 g mol^−1^) (Sigma Aldrich).
  e. Weigh 0.48 g of CuSO_4_ × 5H_2_O (molar mass 249.68 g mol^−1^) (Sigma Aldrich).
  f. Weigh 0.12 g of H_3_BO_3_ (molar mass 61.83 g mol^−1^) (Sigma Aldrich).
  g. Weigh 0.3 g of MnSO_4_ (molar mass 169.02 g mol^−1^) (Sigma Aldrich).
  h. Dissolve in a final volume of 54 mL of MilliQ water.
  i. Add 6 mL of HCl (6 N) (Sigma Aldrich).

***Note:*** Trace element stock for MJ9 medium can be stored for several months in frozen aliquots at −20 °C.

***Note:*** Trace Element Stock is not required when using the LB or M9 medium protocols

### Preparation of LB medium for bacterial growth

**Timing: 4 h**

9. Prepare Lysogeny Broth (LB) medium (calculations refer to 1 L final volume).
  a. Weigh 25 g of LB broth powder (Miller) (Sigma Aldrich).
  b. Dissolve the powder in a final volume of 1 L adding MilliQ water.
  c. Sterilize in autoclave (121 °C, 20 min).
10. Prepare LB-agar plates for bacterial cell culture (calculations refer to 250 mL final volume).
  a. Weigh 6.25 g of LB broth powder (Miller) (Sigma Aldrich) and 3.75 g of agar (Sigma Aldrich).
  b. Add 250 mL of MilliQ water and dissolve the powder mixture (agar will completely dissolve only after heating during autoclave sterilization – see next point).
  c. Sterilize in autoclave (121 °C, 20 min).
  d. Allow the sterilized LB-agar mixture to cool down to ∼ 50°C.
  e. Add 500 μL of 50 mg mL^−1^ ampicillin stock solution.
    **CRITICAL:** Antibiotics must be added after cooling down to ∼ 50 °C to prevent thermal degradation.
  f. Pour the LB-agar into the Petri dishes under aseptic conditions inside the laminar flow cabinet.
  g. Wait until the agar solidifies and seal the plate with parafilm.
  h. Store the LB-agar plates at 4 °C upside down, to prevent aqueous vapor condensation on the surface of the LB-Agar.
    ***Alternatives:*** to obtain higher yields (up to 120 mg L^−1^ fermentation), other rich media (*i.e.* Super Broth, Research Products International) can be used in place of LB.

### Preparation of medium for production of ^15^N-, ^13^C- or ^15^N,^13^C-enriched 3CL^pro^

**Timing: 1 h**

11. MJ9 medium in H_2_O. For ^15^N- (or ^15^N,^13^C-) enriched protein samples production in MJ9 medium, prepare MJ9 minimal medium as follows (calculations refer to 1 L final volume):
  a. Weigh 6 g of K_2_HPO_4_ (J.T.Baker).
  b. Weigh 9 g of KH_2_PO_4_ (J.T.Baker).
  c. Weigh 1.5 g of (NH_4_)_2_SO_4_ or (^15^NH_4_)_2_SO_4_ (Sigma Aldrich).
  d. Weigh 0.5 g of sodium citrate dihydrate (J.T.Baker).
  e. Weigh between 3 and 5 g of D-Glucose or D-Glucose (U-^13^C_6_ 99 %) (Sigma Aldrich, Cambridge Isotope).
  f. Dissolve the powder in a final volume of 1 L adding MilliQ water.
  g. Adjust the pH to 6.8.
  h. Sterile filter.
  i. Add 1 mL of vitamin stock (MEM Vitamin Solution 100× – Sigma Aldrich M6895).
  j. Add 1 g of MgSO_4_ × 7H_2_O (J.T. Baker 2500-01).
  k. Add 1 mL of trace elements stock.
  l. Add 1 mL of stock ampicillin.
    ***Alternatives:*** M9 medium. For ^15^N- (or ^15^N,^13^C-) enriched protein sample production in M9 medium, prepare M9 minimal medium as follows (calculations refer to 375 mL final volume):
  m. Weigh 2.25 g of Na_2_HPO_4_ (Sigma Aldrich).
  n. Weigh 1.12 g of KH_2_PO_4_ (J.T. Baker).
  o. Weigh 0.47 g of (NH_4_)_2_SO_4_ or (^15^NH_4_)_2_SO_4_ (Sigma Aldrich).
  p. Weigh 0.09 g of MgSO_4_ (Sigma Aldrich).
  q. Weigh 0.19 g of NaCl (Sigma Aldrich).
  r. Weigh between 1.2 and 1.9 g of D-Glucose or D-Glucose (U-^13^C_6_ 99 %) (Sigma Aldrich, Cambridge Isotope).
  s. Dissolve the powder in a final volume of 1 L by adding MilliQ water.
  t. Sterile filter.

### Preparation of media for production of perdeuterated ^15^N,^13^C, ^2^H-enriched 3CL^pro^

**Timing: 2 h**

12. For production of perdeuterated ^15^N,^13^C,^2^H-enriched protein samples, prepare solutions as follows:
  a. For MJ9 minimal medium A (calculations refer to 50 mL final volume in H_2_O).
    i. Weigh 0.3 g of K_2_HPO_4_ (J.T.Baker).
    ii. Weigh 0.45 g of KH_2_PO_4_ (J.T.Baker).
    iii. Weigh 0.075 g of (^15^NH_4_)_2_SO_4_ (Sigma Aldrich).
    iv. Weigh 0.025 g of sodium citrate dihydrate (J.T.Baker).
    v. Weigh between 0.15 and 0.25 g of D-Glucose (U-^13^C_6_ 99 %) (Cambridge Isotope).
    vi. Dissolve the powder in a final volume of 50 mL adding MilliQ water.
    vii. Adjust the pH to 6.8.
    viii. Sterile filter.
    ix. Add 50 μL of vitamin stock (MEM Vitamin Solution 100× – Sigma Aldrich M6895).
    x. Add 0.05 g of MgSO_4_ × 7H_2_O (J.T. Baker 2500-01).
    xi. Add 50 μL of trace elements stock (1000×).
  b. For MJ9 minimal medium B (calculations refer to 100 mL final volume in ^2^H_2_O).
    i. Weigh 0.6 g of K_2_HPO_4_ (J.T.Baker).
    ii. Weight 0.9 g of KH_2_PO_4_ (J.T.Baker).
    iii. Weigh 0.15 g of (^15^NH_4_)_2_SO_4_ (Sigma Aldrich).
    iv. Weigh 0.05 g of sodium citrate dihydrate (J.T.Baker).
    v. Weigh between 0.3 and 0.5 g of D-Glucose (^13^C_6_ 99 %) (Cambridge Isotope).
    vi. Dissolve the powder in a final volume of 100 mL adding ^2^H_2_O (CortecNet).
    vii. Adjust the pH to 6.8.
    viii. Sterile filter.
    ix. Add 100 μL of vitamin stock (MEM Vitamin Solution 100× – Sigma Aldrich M6895).
    x. Add 0.1 g of MgSO_4_ × 7H_2_O (J.T. Baker 2500-01).
    xi. Add 100 μL of trace elements stock (1000×).
  c. For MJ9 minimal medium C (calculations refer to 500 mL final volume in ^2^H_2_O).
    i. Weigh 3.0 g of K_2_HPO_4_ (J.T.Baker).
    ii. Weigh 4.5 g of KH_2_PO_4_ (J.T.Baker).
    iii. Weigh 0.75 g of (^15^NH_4_)_2_SO_4_ (Sigma Aldrich).
    iv. Weigh 0.25 g of Sodium citrate dihydrate (J.T.Baker).
    v. Weigh between 1.5 and 2.5 g of D-Glucose (U-^13^C_6_ 99 %; 1,2,3,4,5,6,6-D7, 97–98 %) (Sigma Aldrich, Cambridge Isotope).
    vi. Dissolve the powder in a final volume of 500 mL adding ^2^H_2_O (CortecNet).
    vii. Adjust the pH to 6.8.
    viii. Sterile filter.
    ix. Add 500 μL of vitamin stock (MEM Vitamin Solution 100× – Sigma Aldrich M6895).
    x. Add 0.5 g of MgSO_4_ × 7H_2_O (J.T. Baker 2500-01).
    xi. Add 500 μL of trace elements stock (1000×).

### SUMO protease expression and purification

**Timing: 6 days**

Cell transformation and DNA extraction are performed following the protocol described in the section transformation of Escherichia coli cells using plasmid pGTM_YR375_SUMO_Protease_001 instead of plasmid pGTM_CoV2_NSP5_004_SUMO. Expression of His_8_-MBP-SUMO^pro^, as well as cell lysis, soluble extract recovery, and a first step of immobilized metal affinity chromatography (IMAC) are performed following steps 1–3 as described in the section expression and purification of SARS-CoV-2 3CLpro, using lysis, washing, and elution buffers at pH 7.5 instead of pH 8.0. The His_8_-MBP-SUMO^pro^ eluted from the IMAC is buffer-exchanged in a storage buffer containing 50 mM Tris-HCl, 150 mM NaCl, 10 % glycerol, 0.5 mM DTT, at adjusted pH 7.5; the expressed protease retains its proteolytic activity with no need for cleavage of the His_8_-MBP tag by TEV protease. It can be concentrated up to 1 mg mL^−1^ and stored in 1 mL aliquots at −80 °C. It will be used at a ratios (w/w) of 1:1000 to 1:100 to cleave the His_6_-SUMO tag from His_6_-SUMO-3CL^pro^, to obtain native 3CL^pro^.

***Note:*** His_8_-MBP-SUMO^pro^ has a pI of ∼ 10, and its purification protocol uses a buffer at pH 7.5. However, for His_6_-SUMO-3CL^pro^ (pI ∼ 5.8) and 3CL^pro^ (pI ∼ 5.9), the purification protocol (described below) used a buffer at pH 8.0, which is farther from the isoelectric points of these constructs.

***Note:*** Specific activity of His_8_-MBP-SUMO^pro^ may vary depending on the protein batch preparation, and the optimal ratio and cleavage temperature should be empirically determined by performing cleavage assays on small scale samples. In our experience, undergoing multiple freeze/thaw cycles can reduce protease activity. Therefore, we recommend freezing the purified SUMO^pro^ in small (*e.g.* 50 μL) aliquots, which can be stored for up to 3 months. Individual aliquots should be thawed immediately before needed in the 12–16 h cleavage/dialysis.

#### Transformation of *Escherichia coli* cells

**Timing: 18 h**

13. Transformation of XL10 Gold cells by heat shock for DNA propagation purposes^9^ (Day 1).
  ***Note:*** Additions of DNA and liquid medium to the cell suspension, as well as transferring and plating procedures, must be carried out working under aseptic conditions inside the laminar flow cabinet or using a Bunsen burner to prevent cell culture contamination.
  a. Pre-warm 1 mL of Super Optimal broth with Catabolite repression (SOC) medium at 37 °C in a 1.5 mL sterile microcentrifuge tube.
  b. Thaw one aliquot (50–100 μL) of XL10 Gold *Escherichia coli* cells on ice.
  c. Pipette *ca.* 50 ng of the pGTM_CoV2_NSP5_004_SUMO plasmid directly into the bacterial suspension.
  d. Mix by gently flicking the tube and incubate on ice for 30 min.
  e. Perform the heat shock by transferring the suspension at 42 °C (use either a dry or water bath) for 60 s and then on ice for 2 min.
  f. Add the pre-warmed SOC medium to the suspension and incubate for 45 min at 37 °C under agitation (at 225 rpm).
  g. Plate 100 μL of the suspension on a previously prepared LB-agar Petri dish with 100 μg mL^−1^ ampicillin.
  h. Incubate at 37 °C for 16 h under agitation (at 225 rpm).
14. Plasmid propagation and DNA extraction from XL10 Gold cells (Days 2 and 3).
  a. Pick a single colony from the plates prepared in step 13 and containing transformed XL10 Gold cells, inoculate it in 5 mL of SOC or LB medium with 100 μg mL^−1^ ampicillin and incubate at 37 °C for 16 h under agitation (at 225 rpm).
  b. Carry out the DNA extraction by using a NucleoSpin Plasmid (NoLid) kit (Macherey-Nagel) and following manufacturer’s instructions (https://www.mn-net.com/media/pdf/45/51/02/Instruction-NucleoSpin-Plasmid.pdf, page 15).
  c. Plasmid DNA can be stored in aliquots at −20 °C.

***Alternatives:*** Other commercially available plasmid extraction kits may also be used.

15. Transformation of BL21(DE3) *Escherichia coli* cells by heat shock for protein expression purposes is carried out using the pGTM_CoV2_NSP5_004_SUMO plasmid extracted from XL10 Gold cells in the previous step, and following the protocol outlined in step 13 of this section using instead BL21(DE3) *Escherichia coli* cells. This will provide transformed BL21(DE3) cells in a plate that can be used for the next part of the protocol.

## Key resources table

**Table:** 

REAGENT or RESOURCE	SOURCE	IDENTIFIER
**Bacterial strains**		

XL10-Gold Ultracompetent Cells	Agilent Technologies	200317
BL21(DE3) Competent Cells	Agilent Technologies	200131

**Chemicals, peptides, and recombinant proteins**		

Ulp1 Sumo Protease	This work	Described above
Ampicillin sodium salt	Sigma-Aldrich	A9518
Dithiothreitol (DTT)	Sigma-Aldrich	D9779
Isopropyl-β-D-thiogalactopyranoside (IPTG)	Sigma-Aldrich	I6758
Tris(2-carboxyethyl)phosphine hydrochloride (TCEP)	Sigma-Aldrich	C4706
Super Optimal Broth with catabolite repression (SOC) medium	Sigma-Aldrich	S1797
LB broth powder (Miller)	Sigma-Aldrich	L3522
Super broth powder	Research Products International	S33050
Agar	Sigma-Aldrich	A1296
Dnase I	Sigma-Aldrich	DN25
Magnesium chloride (MgCl_2_)	Sigma-Aldrich	M8266
Sodium chloride (NaCl)	Sigma-Aldrich	S9888
Trizma base (Tris)	Sigma-Aldrich	T6066
Imidazole	Sigma-Aldrich	I2399
Ethylenediaminetetraacetic acid (EDTA)	Sigma-Aldrich	E9884
Iron(III) chloride hexahydrate (FeCl_3_ × 6H_2_O)	Sigma-Aldrich	236489
Zinc sulfate heptahydrate (ZnSO_4_ × 7H_2_O)	Sigma-Aldrich	221376
Cobalt(II) chloride hexahydrate (CoCl_2_ × 6H_2_O)	Sigma-Aldrich	255599
Sodium molybdate dihydrate (Na_2_MoO_4_ × 2H_2_O)	Sigma-Aldrich	M1003
Copper(II) sulfate pentahydrate (CuSO_4_ × 5H_2_O)	Sigma-Aldrich	209198
Boric acid (H_3_BO_3_)	Sigma-Aldrich	B0394
Manganese(II) sulfate (MnSO_4_)	Sigma-Aldrich	M7506
Hydrochloric acid (HCl)	Sigma-Aldrich	XX0628
Sodium phosphate monobasic (NaH_2_PO_4_)	Sigma-Aldrich	74092
Potassium phosphate monobasic (KH_2_PO_4_)	J.T.Baker	3246-05
Potassium phosphate dibasic (K_2_HPO_4_)	J.T.Baker	3252-01
Sodium citrate dihydrate (NaC_6_H_5_O_7_ × 2H_2_O)	J.T.Baker	3646-01
Magnesium sulfate heptahydrate (MgSO_4_ × 7H_2_O)	J.T.Baker	2500-01
MEM Vitamin Solution (100×)	Sigma-Aldrich	M6895
^15^N-ammonium sulfate (^15^NH_4_)_2_SO_4_	Sigma-Aldrich	299286
^12^C D-Glucose	Sigma-Aldrich	G7520
D-Glucose (U-^13^C_6_,99 %; 1,2,3,4,5,6,6-D7, 97–98 %)	Cambridge Isotope	CLM-1396-5
Deuterium oxide (^2^H_2_O)	CortecNet	CD5251P1000
Nickel(II) sulfate (NiSO_4_)	Sigma-Aldrich	656895

**Critical commercial assays**		

NucleoSpin Plasmid NoLid	Macherey-Nagel	740499.50
Dabsyl-KTSAVLQ/SGFRKME-EDANS	GenScript Inc	Custom Synthesis
5(2Aminoethylamino)1Napthalene sulfonic acid (EDANS)	MedChemExpress	ICN15474001

**Recombinant DNA**		

pGTM_CoV2_NSP5_004_SUMO	This work – available from Addgene	AddGene ID: 190062
pGTM_CoV2_NSP5_C145A_001_SUMO	This work – available from Addgene	AddGene ID: 192349
pGTM_YR375_SUMO_Protease_001	This work – available from Addgene	AddGene ID: 190063

**Other**		

Sonicator 3000	Misonix	S-3000/R2225
Amicon Ultra Centrifugal Filter Unit (MWCO 10000 Da)	Merck Millipore	UFC90110
5-mL HiTrap Chelating HP	Cytiva	17040901
HiPrep 26/10 Desalting	Cytiva	17508701
HiLoad® 16/60 Superdex® 75	Cytiva	28988946/17104401
Infinite M1000 PRO microplate reader	TECAN	30063849/140100256
Jupiter C4 column	Phenomenex	00F-4167-B0
ESI-Q-TOF spectrometer	Xevo, Waters	N/A
Shigemi 5 mm Symmetrical NMR microtube	Shigemi, INC	BMS-005B
Bruker INOVA 700 MHz NMR spectrometer	Bruker	N/A
EasyXtal 15-well crystallization plate	NeXtal	132006 132006
Protein Crystallization Cabinet	RUMED	PKS-E100
Magnetic Cryo Vials	Molecular Dimensions	MD7-402
SPINE Sample Changer Basket	Molecular Dimensions	MD7-510
Magnetic Mounted LithoLoops (0.20 mm)	Molecular Dimensions	MD7-136
Magnetic Cryo wand	Molecular Dimensions	MD7-411

**Software and algorithms**		

NMRPipe	Delaglio, F. et al. (1995)^10^	https://www.ibbr.umd.edu/nmrpipe/
POKY	Lee, W. et al. (2021)^11^	https://sites.google.com/view/pokynmr
NMRFAM-SPARKY	Lee, W. et al. (2015)^12^	https://nmrfam.wisc.edu/nmrfam-sparky-distribution/
MXCuBE	Gabaldinho, J. et al. (2010)^13^ Oscarsson, M. et al. (2019)^14^	https://mxcube.github.io/mxcube/
XDS	Kabsch, W. (2010)^15^	https://strucbio.biologie.uni-konstanz.de/xdswiki/index.php/Xds
CCP4	Winn, M. D. et al. (2011)^16^	https://www.ccp4.ac.uk/
AIMLESS	Evans, P. R. and Murshudov, G. N. (2013)^17^	https://www.ccp4.ac.uk/html/aimless.html
REFMAC	Murshudov, G. N. et al. (2011)^18^	https://www.ccp4.ac.uk/html/refmac5/description.html
COOT	Emsley P. and Cowtan K. (2004)^19^	https://www2.mrc-lmb.cam.ac.uk/personal/pemsley/coot/
Magellan™ v 7.2		https://lifesciences.tecan.com/software-magellan
GraphPad Prism		https://www.graphpad.com
Microsoft Excel		https://www.microsoft.com/en-us/microsoft-365/excel

## Materials and equipment

### Lysis buffer at pH 8.0

Prepare fresh before every use by adding DNase and MgCl_2_ to 30 mL of loading/washing buffer (see below)

**Table:** 

Reagent	Final concentration	Amount for 30 mL
Dnase 40 mg mL^−1^	40 μg mL^−1^	30 μL
MgCl_2_ 1 M	10 mM	300 μL

**Table:** Loading / Washing Buffer at pH 8.0 (for immobilized metal affinity chromatography, IMAC)

Reagent	Final concentration	Amount for 1 L
Tris-HCl	20 mM	2.42 g
NaCl	300 mM	17.53 g
Imidazole	10 mM	0.68 g
Dithiothreitol (DTT) 1 M	1 mM	1 mL

Prepare 1 L, adjust to pH 8.0, and store at room temperature (20–25 °C) for up to three days. Pass through 0.22 μm filter and degas before use

**Table:** Elution buffer at pH 8.0 (for IMAC)

Reagent	Final concentration	Amount for 0.5 L
Tris-HCl	20 mM	1.21 g
NaCl	300 mM	8.76 g
Imidazole	300 mM	10.21 g
DTT 1 M	1 mM	0.5 mL

Prepare 0.5 L, adjust to pH 8.0, and store at room temperature (20–25 °C) for up to three days. Pass through 0.2 μm filter and degas before use

**Table:** Size exclusion chromatography (SEC) buffer at pH 7.5

Reagent	Final concentration	Amount for 0.5 L
Tris-HCl	20 mM	1.21 g
NaCl	50 mM	1.46 g
EDTA	1 mM	0.146 g
TCEP 0.1 M	1 mM	5 mL

Prepare 0.5 L, adjust to pH 7.5, and store at 4 °C for up to one week. Pass through 0.2 μm filter and degas before use

**Table:** Förster resonance energy transfer (FRET) buffer at pH 7.3

Reagent	Final concentration	Amount for 0.1 L
Tris-HCl	20 mM	0.242 g
NaCl	100 mM	0.292 g
EDTA	1 mM	0.0292 g
DTT 1 M	1 mM	1 mL
BSA∗	0.1 mg mL^−1^	

Prepare 0.1 L, adjust to pH 7.3, and store at 4 °C

∗To be added just before setting up the assay

## Step-by-step method details

### Expression and purification of SARS-CoV-2 3CL^pro^

**Timing: 4 days**

This section details all the experimental steps needed to express His_6_-SUMO-3CL^pro^ from BL21(DE3) *Escherichia coli* cells harboring the pGTM_CoV2_NSP5_004_SUMO (or pGTM_CoV2_NSP5_C145A_SUMO) plasmid, and to obtain 3CL^pro^ (or C145A-3CL^pro^) in a pure and native form.

1. Expression of His_6_-SUMO-3CL^pro^ by induction with IPTG of transformed BL21(DE3) cells (days 1 and 2):
  ***Note:*** The transferring of cell cultures, as well as the addition of reagents and the collection of cell samples for the measurement of cellular growth must be carried out working under aseptic conditions inside the laminar flow cabinet or using a Bunsen burner to prevent cell culture contamination.
  a. Prepare a starter culture of transformed BL21(DE3) cells by picking and inoculating a single colony (from plates prepared at step 13 in the before you begin section, which contain transformed BL21(DE3) cells) in 50 mL of LB broth with 100 μg mL^−1^ ampicillin added. Incubate the resulting suspension for 16 h at 37 °C under agitation (at 225 rpm).
  b. Transfer the starter culture to 1 L of LB broth with 100 μg mL^−1^ ampicillin added and incubate the suspension at 37 °C under agitation (225 rpm).
  c. Measure the optical density at 600 nm (OD_600_) every 30 min until it reaches *ca.* 0.6 units (approximately 1.5–2 h).
  d. Add IPTG to a final concentration of 1 mM to induce protein expression.
  e. Incubate for 16 h at 17 °C under agitation (225 rpm).
    ***Alternatives:*** To prepare isotopically-enriched samples for NMR experiments using MJ9 minimal medium optimized for isotope-enrichment,^8^ at the end of point a above, transfer 25 mL of the obtained starter culture to 1 L of MJ9 minimal medium added with 50 μg mL^−1^ ampicillin. The resulting suspension should be incubated at 37 °C under stirring (225 rpm) and treated as described from point c onwards.
    ***Alternatives:*** While the best results are usually obtained using MJ9 medium, M9 minimal medium can also be used. In this case, once OD_600_ reaches *ca.* 0.6 units (point c above), harvest the cells by centrifugation for 20 min at 7000 × *g* at room temperature and resuspend the pellet in 375 mL of M9 minimal medium containing ^15^N ammonium sulphate (and/or ^13^C-enriched glucose) added with 50 μg mL^−1^ ampicillin. Incubate the resulting suspension for 20 min at 17 °C under agitation (225 rpm), then induce and further incubate as described in points d and e.
    ***Alternatives:*** To prepare ^15^N,^13^C,^2^H-enriched samples for NMR experiments using MJ9 minimal medium optimized for isotope-enrichment,^8^ fully replace step 1 a-e with the following procedure:
  f. Prepare a starter culture of transformed BL21(DE3) cells by picking and inoculating a single colony (from plates prepared at step 13 in the before you begin section) in 1.0 mL of LB medium added with 100 μg mL^−1^ ampicillin. Incubate the resulting suspension at 37 °C until OD_600_ reaches 0.6 units (approximately 1.5–2 h).
  g. Harvest the cells by centrifugation (7000 × *g*) at room temperature.
  h. Resuspend the cell pellet in 3.0 mL of MJ9 medium A added with 50 μg mL^−1^ ampicillin.
  i. Incubate the cell suspension in a 15 mL Falcon Tube at 37 °C under agitation (225 rpm) until OD_600_ reaches 0.5 units (approximately 1.5–2 h).
    **CRITICAL:** OD_600_ must not exceed 0.65 units to ensure that the culture is still in the log phase.
  j. Prepare four separate dilutions (1:40, 1:100, 1:200, 1:400) of the cells grown in MJ9 medium A in a final volume of 20 mL using MJ9 medium B (with 50 μg mL^−1^ ampicillin prepared in ^2^H_2_O added).
  k. Incubate the cell suspensions for 16 h at 37 °C under agitation (225 rpm).
  l. Measure the OD_600_ of the cultures and select the one with OD_600_ closest to 0.5 units.
  m. Harvest the cells of the selected culture by centrifugation (7000 × *g*) at room temperature (20–25 °C) for 5 min.
  n. Resuspend the cell pellet in 500 mL of MJ9 medium C with 50 μg mL^−1^ ampicillin prepared in ^2^H_2_O added.
  o. Incubate the cell suspension at 37°C under agitation (225 rpm) until OD_600_ reaches 0.35 units, then further incubate for 45 min at 17 °C under agitation (225 rpm).
  p. Induce protein expression with 1 mM IPTG (using IPTG stock prepared in ^2^H_2_O).
  q. Incubate the culture for 12–16 h at 17 °C under agitation (225 rpm).
2. Cell lysis and soluble extract recovery (Day 3).
  a. Harvest the cells by centrifuging the culture for 30 min at 7000 × *g* at 4 °C.
  b. Gently resuspend the harvested cells in 20 mL lysis buffer.
  c. Lyse cells by sonication (50 % power, 5 s/5 s on/off, for a total on time of 10 min) on ice.
  d. Collect a 10 μL aliquot of the lysate (total lysate, TL) in a 1.5 mL microcentrifuge tube for SDS-PAGE analysis; add the appropriate amount of loading dye (usually 5 μL or what is recommended by manufacturer) and freeze at −20 °C.
  e. Centrifuge the lysate for 30 min at 17000 × *g* at 4 °C to separate the soluble extract from the insoluble proteins and cell debris: collect the soluble extract. Collect 10 μL aliquots each of the soluble extract (S) and the insoluble pellet (I) in 1.5 mL microcentrifuge tubes for SDS-PAGE analysis, add the loading dye to each tube, and freeze at −20 °C. Discard the remaining insoluble portion according to standard biosafety protocols.
  f. Filter the soluble extract using 0.45 μm syringe filters and store at 4 °C.

***Alternatives:*** Lysis can also be carried out by using a French press (SLM Aminco). The harvested cells can be resuspended in *ca.* 25 mL of lysis buffer and homogenized by using a Potter-Elvehjem tissue grinder to obtain a homogeneous cell suspension and to disrupt any solid salt precipitate possibly formed during cellular growth. The resuspended cells are lysed performing three lysis cycles using the French press at 20,000 psi (1 psi = 6.9 kPa).

**CRITICAL:** While lysing, both procedures cause an abrupt temperature increase in the cell suspension. It is highly recommended to keep the lysate on ice and to use a container with good thermal conductivity during the procedure to prevent thermal protein denaturation that would negatively affect final protein yield.

3. Immobilized metal affinity chromatography (IMAC) (Day 3).
  ***Note:*** The expressed SARS-CoV-2-3CL^pro^ contains a His_6_-SUMO tag at its N-terminus that allows for protein purification using immobilized nickel affinity chromatography carried out using an ÄKTAprime system with a 5 mL HiTrap Chelating HP column (Cytiva) working at a flow rate of 4 mL min^−1^.
  ***Note:*** Due to the high amount of His_6_-SUMO-3CL^pro^ expressed from 1 L of cell culture, it is recommended to split the soluble fraction and perform the IMAC in two steps to prevent column overloading and possible loss of protein. Two or more columns placed in series can also be used to achieve high amounts of protein recovery and speed up the purification procedure.
  a. Wash the 5 mL HiTrap Chelating HP column with five column volumes (CVs) of MilliQ water and regenerate with 0.1 M NiSO_4_ following the manufacturer’s protocol https://cdn.cytivalifesciences.com/api/public/content/digi-11225-pdf).
  b. Equilibrate the column with 5 CVs of washing buffer.
  c. Load the soluble extract onto the column either through a superloop (Cytiva) (using washing buffer) or directly with pump A.
  d. Wash the column with washing buffer and collect the unbound material (hereafter called Flow-Through, FT). Monitor the absorbance at 280 nm (Abs_280_) and wait until the baseline returns to zero (typically *ca.* 10–20 CVs) to ensure complete removal of the FT.
  e. Collect a 10 μL aliquot of FT in a 1.5 mL microcentrifuge tube for SDS-PAGE analysis, add the loading dye, and freeze at −20 °C. Store the rest of FT at 4 °C.
  f. Elute the bound proteins by increasing the imidazole concentration by setting a linear gradient from 0 to 100 % of elution buffer in ten CVs and collecting eluate in 2 mL fractions. A representative chromatogram is shown in Figure 3A.

**Figure 3:**
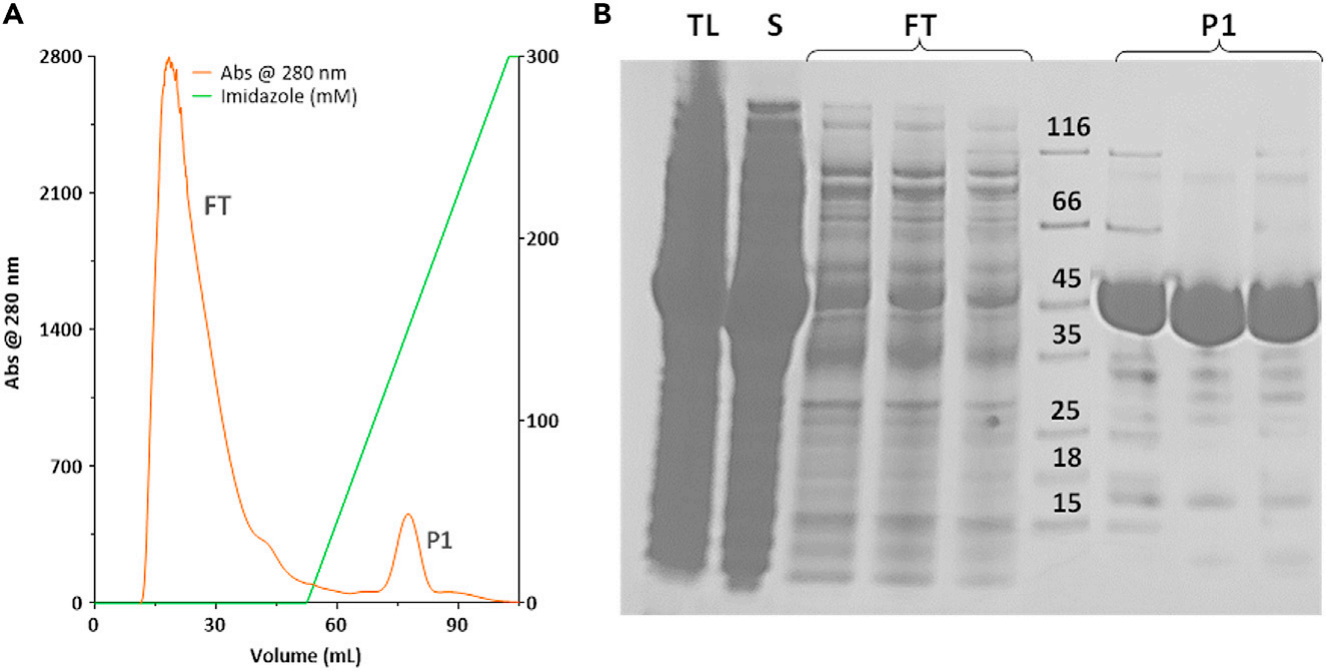
Immobilized metal (nickel) affinity chromatography (IMAC) (A) Representative chromatogram showing the entire process of protein loading, flow through collection, and protein elution. (B) SDS – PAGE showing total lysate (TL), soluble extract (S), flowthrough (FT) and fractions eluted in peak 1 (P1) of the IMAC. His_6_-SUMO-3CL^pro^ (molar mass of *ca*. 46 kDa) is detected in lanes corresponding to peak P1.
  g. Collect a 10 μL aliquot of each fraction in 1.5 mL microcentrifuge tubes for SDS-PAGE analysis, add the loading dye and freeze at −20 °C.
  h. Once the elution is complete, wash the column with five CVs of elution buffer, MilliQ water, and 20 % v/v ethanol each to return the column in storing conditions. Store the column at 4 °C.
  i. Analyze the total lysate (TL), the soluble extract (S), as well as the FT and the fractions eluted from the IMAC, for protein presence and purity by running an SDS-PAGE loading 5 μL of the samples previously collected and boiled for 5 min at 90 °C (a representative protein gel is shown in Figure 3B).
  j. Pool the fractions containing His_6_-SUMO-3CL^pro^ and store at 4 °C.
4. Cleavage of the His_6_-SUMO tag by SUMO protease and buffer exchange (Day 3).
  ***Note:*** The proteolytic cleavage of the N-terminal His_6_-SUMO tag is performed by the addition of His_8_-MBP-SUMO^pro^ to the expressed His_6_-SUMO-3CL^pro^. General protocols for SUMO protease cleavage recommend carrying out cleavage in the presence of imidazole concentrations lower than 150 mM to prevent adverse effects of imidazole on the activity of SUMO protease. His_6_-SUMO-3CL^pro^ elutes at *ca*. 180 mM imidazole concentration from the IMAC, therefore a buffer exchange may be useful to decrease the concentration of imidazole in the protein solution prior to treating with SUMO protease. However, uncontrolled cleavage of His_6_-SUMO tag from His_6_-SUMO-3CL^pro^, probably by trace amounts of native *E. coli* proteases, has been observed prior to the addition of SUMO protease. This can be minimized by carrying out cleavage in imidazole immediately after purifying the His_6_-SUMO-3CL^pro^ fusion, with minimal to no negative effects on the production of pure and native 3CL^pro^ (as described in this protocol). Nevertheless, if SUMO protease activity issues are observed, a buffer exchange into the Loading / Washing Buffer can be carried out before treatment with SUMO protease by performing points c-e before points a and b of the following procedure. Buffer exchange can be carried out using an ÄKTAprime system and a HiPrep 26/10 Desalting column (Cytiva) working at a flow rate of 10 mL min^−1^, or by dialysis. In any case, buffer exchange is mandatory to decrease the concentration of imidazole in the protein prior to the following protocol for removal of the cleaved tag and the SUMO protease from the native 3CL^pro^ by reverse IMAC.
  ***Note:*** Incubation times for complete cleavage of N-terminal His_6_-SUMO tag by SUMO protease may vary depending on protease activity. As described in the before you begin section, the optimal ratio and cleavage temperature (4 or 26 °C) should be empirically determined by performing cleavage assays on small-scale samples. In this protocol a 1:100 (w/w) ratio of SUMO protease generally provides > 99 % cleavage after 3 h when carried out in a pH 8.0 buffer containing reducing agent (*e.g.* the Elution Buffer) at 26 °C.
  a. Collect a 10 μL aliquot of the pre-cleaved His_6_-SUMO-3CL^pro^ in a 1.5 mL microcentrifuge tube for SDS-PAGE analysis, add the loading dye, and freeze at −20 °C.
  b. Add 1:100 (w/w) ratio of His_8_-MBP-SUMO^pro^ (ULP1) to the protein solution (estimated by absorbance at 280 nm and considering ε_280_ = 100,855 M^−1^ cm^−1^ and 32,890 M^−1^ cm^−1^ for His_8_-MBP-SUMO^pro^ and His_6_-SUMO-3CL^pro^, respectively) in Elution Buffer (or after buffer exchange in Loading / Washing Buffer to reduce imidazole concentrations, if preferred) and incubate for 3 h at 26°C to ensure complete cleavage of the N-terminal tag.
  c. During this hydrolysis incubation, wash the HiPrep 26/10 Desalting column (Cytiva) with five CVs of MilliQ water and equilibrate it with five CVs of Loading / Washing Buffer.
  d. At the end of the hydrolysis incubation, load up to 10 mL of protein solution onto the HiPrep 26/10 Desalting column using a sample loop of the appropriate volume, and carry out the buffer exchange and protein elution using Loading / Washing Buffer, monitoring Abs_280_ and stopping sample collection when Abs_280_ returns to baseline.
    ***Note:*** Imidazole elution strongly affects the conductance of the eluate. Therefore, it is recommended to monitor the change of this parameter value during elution to ensure the correct separation from the protein solution (to read more about the basis of desalting procedures and technical details using HiPrep 26/10 Desalting visit manufacturer’s web site at https://www.cytivalifesciences.com/).
  e. Complete washing of the Desalting column with five CVs of washing buffer, MilliQ water, and 20 % v/v ethanol each to return the column in storing conditions. Store the column at 4 °C.
    ***Alternatives:*** Cleavage of His_6_-SUMO tag from 3CL^pro^ and buffer exchange can also be carried simultaneously by dialyzing the protein eluted from the IMAC in the presence of a 1:100 (w/w) ratio SUMO protease, for 12–16 h at 4 °C, against Loading / Washing Buffer prepared without imidazole (*i.e.*, 20 mM Tris pH 8.0, 300 mM NaCl, 1 mM DTT).
5. Removal of the cleaved tag and SUMO protease from cleaved 3CL^pro^ solution (Day 4).
  ***Note:*** The cleaved His_6_-SUMO tag and His_8_-MBP-SUMO^pro^ both present N-terminal His-tags allowing for separation from the native 3CL^pro^ through an additional step of IMAC. Cleaved 3CL^pro^ will not interact with the column resin and will therefore be collected in the flowthrough, while His_6_-SUMO tag and His_8_-MBP-SUMO^pro^ will bind to the column and will be eluted at a higher imidazole concentration. Separation is carried out using an ÄKTAprime system and a 5 mL HiTrap Chelating HP column (Cytiva) loaded with Ni^2+^, working at a flow rate of 4 mL min^−1^.
  a. Wash the 5 mL HiTrap Chelating HP column (Cytiva) used the day before with five CVs of MilliQ water and equilibrate it with five CVs of Loading / Washing Buffer.
  b. Load the protein solution onto the column using a superloop (Cytiva) or pump A (while using Loading / Washing Buffer).
  c. Wash the column with Loading / Washing Buffer while monitoring the absorbance at 280 nm (Abs_280_) to recover the cleaved, native 3CL^pro^ in the flowthrough. Store the protein solution at 4 °C.
  d. Collect a 10 μL aliquot of protein solution in a 1.5 mL microcentrifuge tube for SDS-PAGE analysis, add the loading dye, and freeze at −20 °C.
  e. Elute the bound proteins remaining on the column using five CVs of 100 % Elution Buffer until the Abs_280_ baseline returns to zero.
  f. Collect a 10 μL aliquot of the eluted proteins in a 1.5 mL microcentrifuge tube for SDS-PAGE analysis, add the loading dye and freeze at −20 °C.
  g. Wash the column with five CVs of MilliQ water and five CVs of 20 % v/v ethanol to return the column in storing conditions. Store the column at 4 °C.
    ***Alternatives:*** To minimize cleavage of His_6_-SUMO tag from 3CL^pro^ by native *E. coli* proteases, an on-column cleavage of His_6_-SUMO tag can be performed directly on the 5 mL HiTrap Chelating HP column prior to eluting with imidazole. This useful shortcut allows to perform IMAC purification, cleavage of His_6_-SUMO tag from 3CL^pro^, and removal of cleaved His_6_-SUMO tag and His_8_-MBP-SUMO^pro^ from native 3CL^pro^ in one step. In the on-column cleavage protocol, follow points a-e of step 3 (***Immobilized metal affinity chromatography***). Then add 1–2 mL of 1 mg mL^−1^ His_8_-MBP-SUMO-^pro^ (1 mL is sufficient for lower yields, *e. g.,* batches fermented in minimal medium, but 2 mL may be needed for higher yield batches). Run one CV of Loading / Washing Buffer to fully load the protease onto the column and allow cleavage by storing the column 12–16 h at 4 °C. The next morning, elute the cleaved 3CL^pro^ with Loading / Washing Buffer and use the Elution Buffer to elute any remaining uncleaved His_6_-SUMO-3CL^pro^, the His_8_-MBP-SUMO^pro^, and the cleaved His_6_-SUMO tag. In this protocol, the target product is also not exposed to high concentrations of imidazole.
6. Size exclusion chromatography (SEC) (Day 4).
  ***Note:*** A final size exclusion chromatographic (SEC) step provides a fully monodisperse and pure 3CL^pro^ sample in a buffer suitable for downstream structural and biochemical studies. This step is carried out using an ÄKTAprime system and a HiLoad® 16/60 Superdex® 75 column (Cytiva), working at a flow rate of 1 mL min^−1^.
  a. Concentrate the protein solution to a final volume ≤ 5 mL using an Amicon Ultra Centrifugal Filter Unit (MWCO 10,000 Da) (Merck Millipore).
    **CRITICAL:** Although not strictly necessary, this concentration step is highly recommended to expedite the following SEC. During this step it is important to monitor the concentration of the protein solution (by measuring the absorbance at 280 nm and considering ε_280_ = 32,890 M^−1^ cm^−1^), which should not exceed 0.8 mM to avoid possible precipitation. If the desired volume of 5 mL is not achievable, split the protein solution into two or more aliquots to be independently treated in the next purification step.
  b. Wash the column with one CV of MilliQ water and equilibrate it with one CV of SEC buffer.
  c. Load up to 5 mL of protein solution using a sample loop of the appropriate volume and carry out the protein elution using SEC buffer (a representative chromatogram is shown in Figure 4A), collecting the eluate in 1 mL fractions and store at 4 °C.

**Figure 4:**
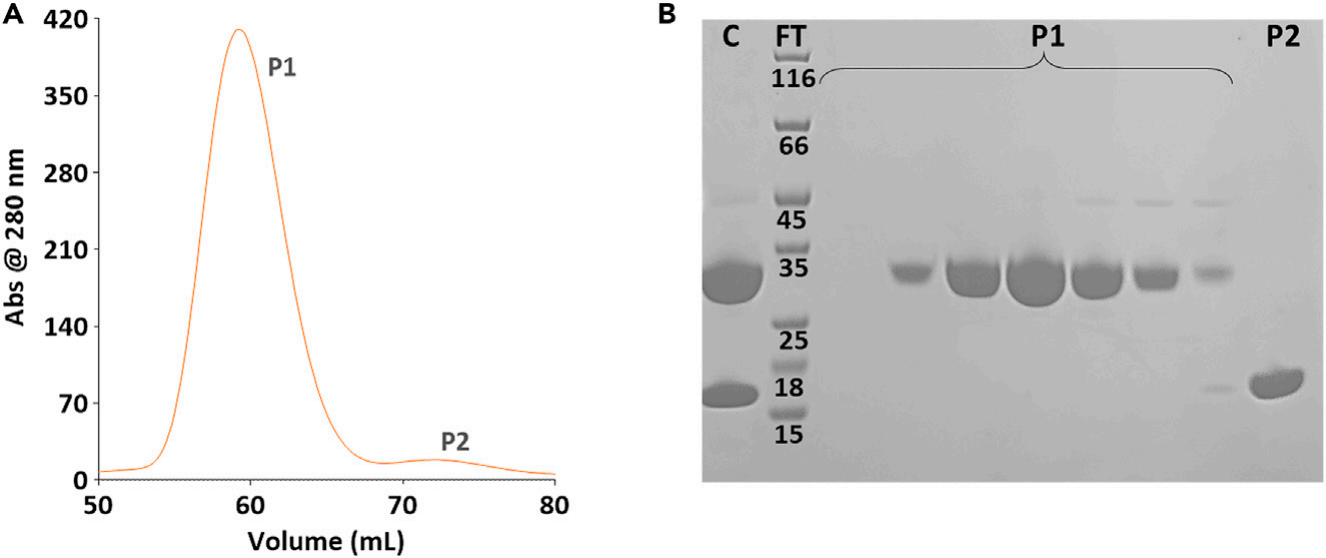
Size exclusion chromatography of native 3CL^pro^ (A) Representative SEC chromatogram and (B) corresponding SDS – PAGE from elution of the SEC column. Lanes C, FT, P1, and P2 correspond respectively to the pre-loaded cleaved protein solution, molecular weight standards, native 3CL^pro^ eluted in peak P1 (at a molar mass of *ca.* 33 kDa for the monomer), and the His_6_-SUMO tag eluted in peak P2 (at a molar mass of *ca.* 12 kDa).
  d. Collect a 10 μL aliquot of each fraction in 1.5 mL microcentrifuge tubes for SDS-PAGE analysis, add the loading dye, and freeze at −20 °C.
  e. Repeat points c - d for each 5 mL of the remaining protein solution.
  f. Upon complete elution of the last protein sample wash the column with one CV of MilliQ water and one CV 20 % v/v ethanol. Store the column at 4 °C.
  g. Analyze all collected fractions for protein presence and purity by running an SDS-PAGE gel loading 5 μL of the samples previously collected and boiled for 5 min at 90°C. Pool the fractions containing native 3CL^pro^. A representative SDS-PAGE is shown in Figure 4B.
  h. Native 3CL^pro^ can be concentrated up to 25 mg mL^−1^ (corresponding to 0.8 mM monomer). At these concentrations, native 3CL^pro^ can be flash-frozen in liquid nitrogen and stored for up to one month at −80 °C.
    ***Note:*** SEC buffer here reported is one among the several buffers in which native 3CL^pro^ can be stored. Buffers most compatible with other downstream studies can be chosen as well.
    ***Alternatives:*** In our experience, the cleaved His_6_-SUMO tag and the His_8_-MBP-SUMO^pro^ can also be effectively separated from the native 3CL^pro^ directly through the SEC procedure, rather than using the HiTrap column. To pursue this alternative, follow the purification procedure until point 4b (***Cleavage of the His***_***6***_***-SUMO tag by SUMO protease***), then skip step 4 points c - e (***Buffer exchange***) and step 5, and go directly to step 6.

### Characterization of SARS-CoV-2 3CL^pro^ by mass spectrometry

**Timing: 3 h**

7. Sample separation by HPLC.
  a. Dilute a 1 mg mL^−1^ solution of 3CL^pro^ 1:10 (v/v) in MilliQ water.
  b. Load the diluted protein sample onto a Jupiter C4 column (Phenomenex) working at a flow rate of 0.4 mL min^−1^ using mobile phases A (H_2_O/formic acid; 100/0.1; v/v) and B (acetonitrile/formic acid; 100/0.1; v/v) to perform a gradient to enhance the separation of the eluting species.
8. Analyze the eluted species using an ESI-Q-TOF spectrometer (Xevo, Waters) using the software provided with the instrument.

**Figure 5:**
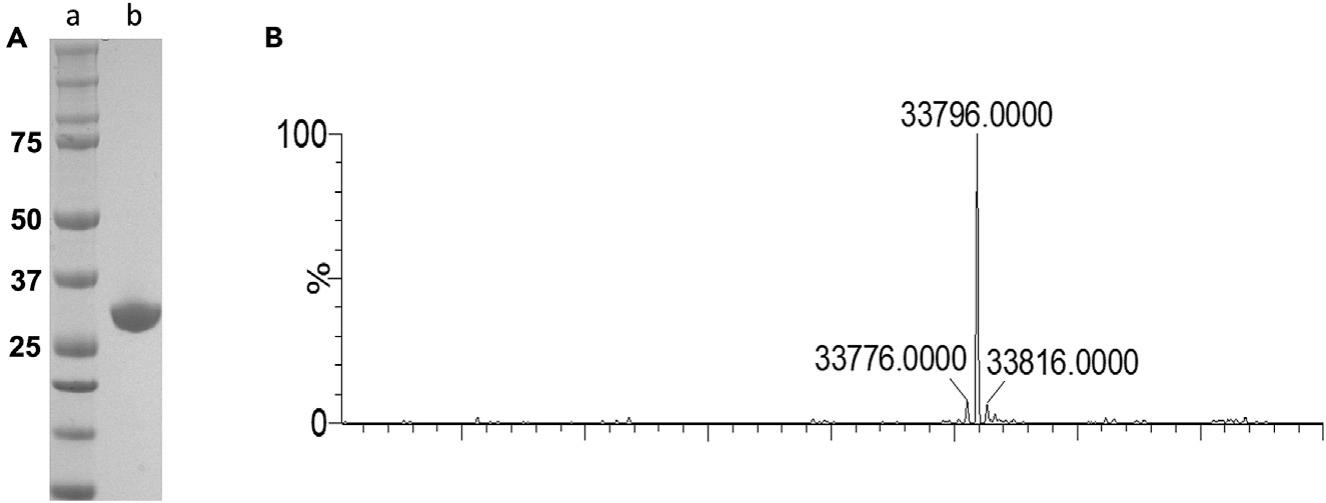
Characterization of purified 3CL^pro^ (A) SDS-PAGE gel showing high quality of SARS-CoV-2 3CL^pro^ sample. Lane a – molecular weight standards; Lane b – SARS-CoV-2 3CL^pro^ post SEC purification (sample prepared as described above in "Size exclusion chromatography (SEC)" section) (B) ESI-MS profile of SARS-CoV-2 3CL^pro^ showing the signal corresponding to an experimental molecular mass of 33,796 Da.

***Note:*** The resulting deconvoluted spectrum shows a single signal corresponding to an experimental molar mass of 33,796 Da (Figure 5). The experimental value perfectly matches that estimated by the amino acid protein sequence (UniProt entry P0DTD1, residues 3264–3569) and establishes that the current protocol for the expression and purification of SARS-CoV-2 3CL^pro^ is a reliable procedure to obtain large quantities of native 3CL^pro^.

### FRET (Förster resonance energy transfer) assay for the characterization of 3CL^pro^ inhibition by IC_50_ determination

**Timing: 2 h**

***Note:*** 3CL^pro^ inhibition properties are characterized by performing FRET assays using peptide substrate KTSAVLQ/SGFRKME (designed with the 3CL^pro^ cleavage site Q/S) labeled with a DABCYL and EDANS FRET pair (fluorophore/quencher) on N and C termini, respectively (DABCYL-KTSAVLQ/SGFRKME-EDANS) (GenScript). Excitation at 360 nm excites the donor EDANS which transfers its energy non-radiatively due to the close proximity of the acceptor (quencher), DABCYL. Upon enzymatic cleavage of the labeled FRET peptide by 3CL^pro^, energy transfer no longer occurs between the fluorophore and quencher, resulting in an increase in fluorescence that can be detected at the emission maximum of the fluorophore EDANS (460 nm). This rate of increase in fluorescence is directly proportional to the enzyme activity. FRET assays are carried out at increasing concentrations of potential 3CL^pro^ inhibitors to screen candidate molecules and evaluate their IC_50_ values (*i.e*., the inhibitor concentration providing a 50 % decrease of enzyme activity).

**Figure 6:**
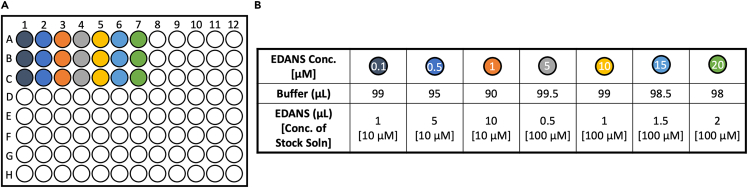
Materials preparation for calibration curve (A) Schematic of 96 microwell plate set-up for FRET calibration curve. (B) Table of reagents for each well.

**Figure 7:**
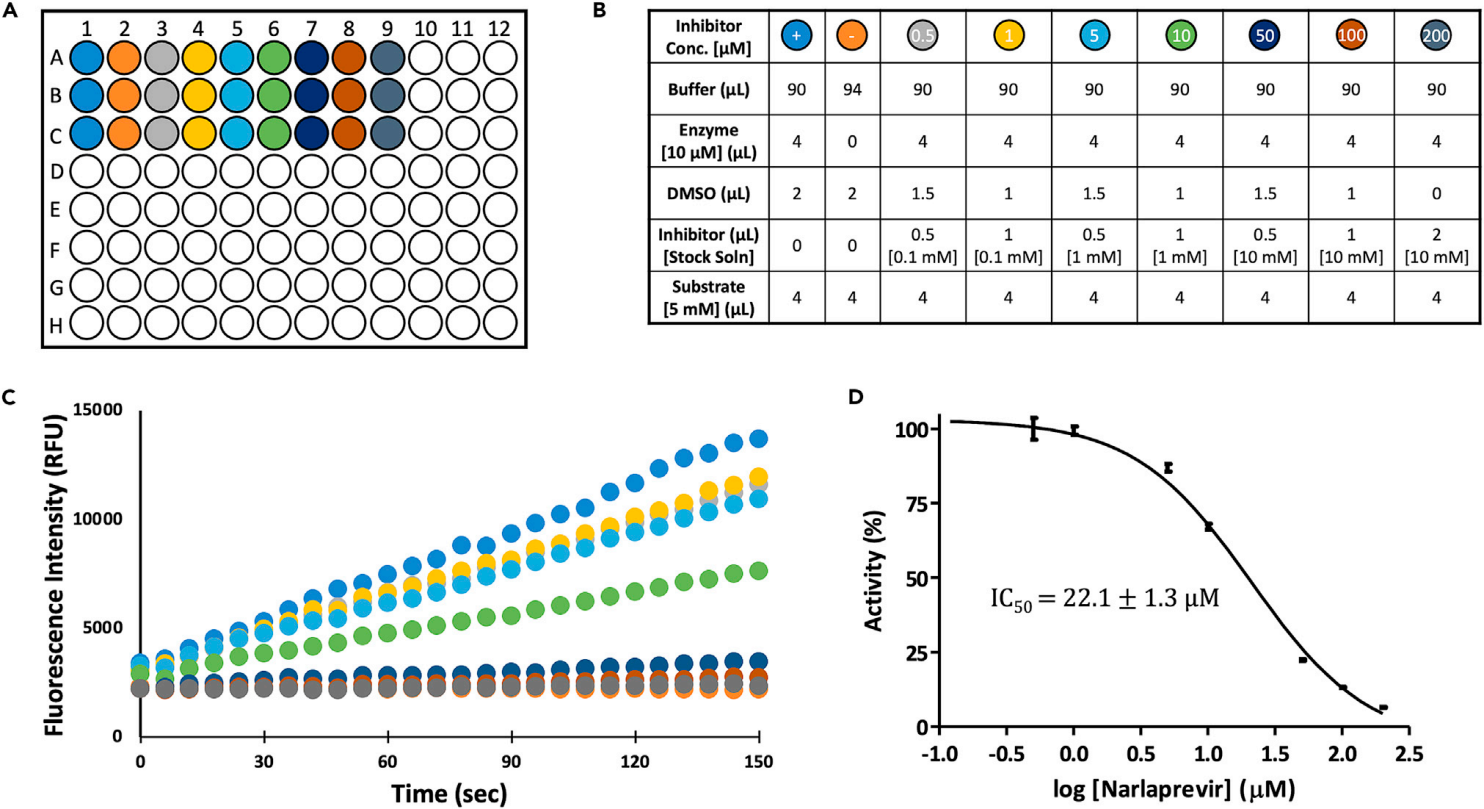
Materials preparation and data processing for IC_50_ determination (A) Schematic of 96-well plate set-up for dose response assay to determine IC_50_. (B) Table of reagents for each well (final DMSO concentration = 4 %). (C) Representative experimental raw data showing the increase of fluorescence intensity as a function of reaction time. (D) Representative dose response curve for Narlaprevir, error bars represent SD's of triplicate measurements.

FRET assays are carried out using black opaque 96-well microplates (Corning) containing a final volume of 100 μL per well. Figures 6 and 7 provide the detailed amounts of reagents placed in each well. Experiments should be run in triplicate and results are monitored and analyzed using a TECAN Infinite M1000 microplate reader and Magellan™ software (or comparable multi-well fluorimeter).

9. Instrument protocol setup:

Use the Magellan™ software of TECAN Infinite M1000 microplate reader with the following parameters: (i) Excitation wavelength: 360 nm; (ii) Emission wavelength: 460 nm. Fluorescence intensity is recorded every 3 s for a total of 15–60 min at room temperature (20–25 °C).

10. Calibration curve for fluorescence-to-activity conversion.
  ***Note:*** Raw data collected during the FRET experiments indicate fluorescence intensity due to substrate proteolysis and are reported in terms of Relative Fluorescence Units (RFU). Preparation of a calibration curve for the conversion of the fluorescence data to cleaved peptide concentration (μM) using 0.10 to 20 μM EDANS is described below:
  a. Prepare a stock solution of 500 μM EDANS (MedChem Express) in DMSO.
  b. Prepare 100 μM and 10 μM dilutions of the 500 μM stock solution using DMSO.
  c. Add FRET buffer to each well according to Figure 6.
  d. Add the appropriate volume of EDANS to each well so that the final concentrations range from 0.10 to 20 μM.
  e. Measure emission at 460 nm using the Magellan™ software of TECAN Infinite M1000 microplate reader.
  f. Plot the measured fluorescence as a function of EDANS concentration in Microsoft Excel or GraphPad Prism.
  g. Perform a linear fit of the data.
    ***Note:*** The resulting best-fit equation will be used to interpolate experimental fluorescence data obtained in the following experiment and convert from RFU per second to substrate hydrolysis rates (μM s^−1^).
    ***Alternatives:*** A calibration curve can also be set up by incubating 200 nM 3CL^pro^ with increasing concentrations of FRET substrate (0.5–50 μM) and monitoring the reaction until the fluorescence signal reaches plateaus for all concentrations, thus indicating that the total amount of the FRET substrate has been cleaved by 3CL^pro^. Final fluorescence values can be plotted as a function of the FRET substrate concentration and fit linearly to generate a calibration curve as described in step 10.
    ***Note:*** For certain FRET studies it is necessary to calibrate the fluorimeter for inner-filter effects arising from UV absorbance by the substrate (or inhibitor) at high concentrations.^20^ However, at the concentrations of substrate described in this protocol, no inner filter effect corrections are required.
11. 3CL^pro^, FRET substrate and inhibitor preparation:
  a. Prepare a 10 mM peptide substrate stock solution in DMSO, and store it as 60 μL aliquots at −20 °C.
  b. Prepare 5 mM peptide substrate samples from the 10 mM stock by diluting 35 μL with an equal volume of FRET buffer.
  c. Prepare inhibitor stocks dissolving them in DMSO and diluting to 10.0, 1.0, and 0.1 mM concentrations in DMSO.
  d. Gently thaw 30 μL of purified 3CL^pro^ on ice. Estimate the protein concentration using Lambert-Beer equation, with ε_280_ = 32,890 A.U. M^−1^ cm^−1^, and dilute the 3CL^pro^ sample to a final concentration of 10 μM using FRET Buffer.
12. Setup of the reaction mixtures in the 96-well microplates:
  a. In each well of the 96-wells microplate add:
    i. FRET buffer as described in Figures 7A and 7B (90 μL of FRET Buffer will be added to every well except for column 2, where a negative control experiment in the absence of enzyme will be set up using 94 μL of FRET Buffer).
    ii. 4 μL of 10 μM 3CL^pro^ stock solution (resulting in 400 nM 3CL^pro^ final concentration) except for the negative control experiment in column 2.
    iii. 0–2.0 μL inhibitor, corresponding to 0–200 μM concentration (refer to Figures 7A and 7B for details).
    iv. Add the appropriate amount of DMSO to maintain the DMSO concentration constant in all wells.
    v. Let the plate equilibrate for 10–15 min at room temperature (20–25 °C).
    vi. Initiate the reaction by adding 4 μL of 5 mM peptide substrate stock (resulting in 200 μM substrate final concentration), and mix by pipetting up and down 2–3 times.
      ***Note:*** The enzymatic reaction begins as soon as the substrate is added to each well. Carefully pipette the substrate into each well, moving as quickly as possible to initiate the FRET data collection (below) with minimal dead time.
  b. Place the 96-well microplate in the Infinite M1000 plate reader (TECAN) and press START on the Magellan software immediately to begin data collection.
  c. Let the reactions run for 10–20 min to measure initial enzyme velocities.
    ***Note:*** Measurements are carried out at saturating substrate concentration (*i.e.* [S] ≫ K_m_ and reaction rate close to V_max_). Final concentration of DMSO = 4 %.
    ***Note:*** Inhibitors vary in their solubility. Therefore, the assay may be adjusted to use final concentrations of DMSO even lower than 1 % for appropriately soluble substrates and inhibitors.
13. Data export and analysis.
  a. Export the data from Magellan in Microsoft Excel .xls or in .csv format.
  b. Analyze the data using GraphPad or other kinetic analysis software:
    i. Plot the fluorescence intensity *vs.* time data (Figure 7C) for each inhibitor concentration.
    ii. Measure the slope of the linear portion of each curve (usually within the first 5 min of reaction) by performing a linear fit on that region.
      ***Note:*** Each obtained slope value corresponds to the initial velocity in the presence of that inhibitor concentration (*V*_*i*_). The slope determined for the positive control measurements in column 1 (Figure 7B), carried out in the absence of inhibitor, corresponds to the initial velocity of the non-inhibited 3CL^pro^ (*V*_*0*_).
    iii. Calculate the average ± standard deviation values for the triplicate measurements carried out at each inhibitor concentration to obtain, for each concentration, an averaged *V*_*i*_ (or *V*_*0*_) value.
    iv. Using the averaged values, calculate residual activity (%) at each inhibitor concentration using the formula
      (Equation 1) $$Residual\;activity\;\left(\%\right)=100\times \frac{V_{i}}{V_{0}}$$
    v. Plot residual activity (%) data as a function of increasing inhibitor concentration and fit them by using the following equation:
      (Equation 2) $$Residual\;activity\;\left(\%\right)=\frac{100}{1+x/{IC}_{50}}$$
    vi. Transform the *x*-axis visualization on a Log_10_ scale to show the plotted experimental data and resulting fit following the typical sigmoidal behavior (Figure 7D).
      ***Note:*** The IC_50_ value estimated by Equation 2 corresponds to the inhibitor concentration at which residual activity is 50 %.
      ***Alternatives:*** This protocol can also be adopted as a method for preliminary screening of a panel of molecules as potential inhibitors by using a single concentration (*e.g.*, 20 μM) of each.

### NMR spectroscopy of 3CL^pro^

**Timing: Approximately 4 h**

**Figure 8:**
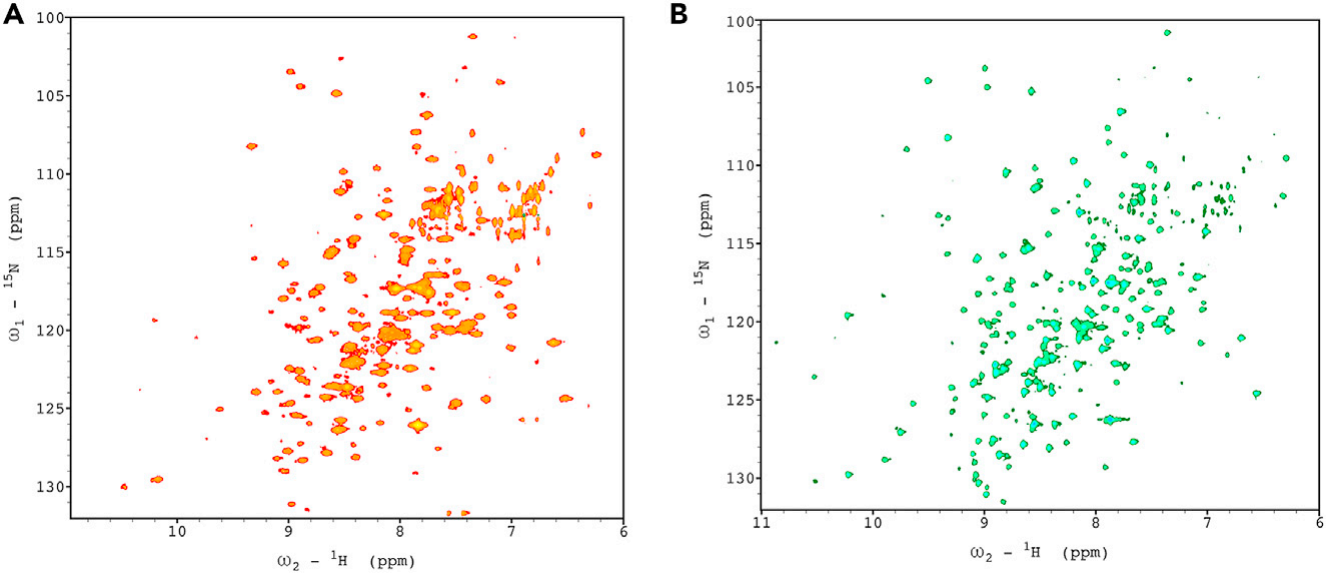
2D ^1^H,^15^N BEST-TROSY NMR spectrum of SARS-CoV-2 3CL^pro^ at 298 K (A) Wild type and (B) [Cys145Ala]- SARS-CoV-2 3CL^pro^ mutant. No significant sample degradation was observed over a 1-week period at room temperature.

14. Sample preparation for the ^15^N NMR experiment.
  a. Gently thaw a 250 μL aliquot of purified 0.8 mM ^15^N-enriched 3CL^pro^ on ice.
  b. Add 25 μL deuterium oxide (CortecNet or Cambridge Isotopes) to the protein solution.
  c. transfer the clear, transparent sample to the Shigemi 5 mm symmetrical NMR microtube (SHIGEMI, INC).
15. Data recording and analysis.
  a. Record ^1^H,^15^N BEST-TROSY spectra of the protein at 298 K on a Bruker 700 MHz NMR spectrometer using 256 increments, 24 scans, and 1.2 s relaxation delay.
  b. Sweep widths of 13 ppm and 40 ppm and offsets of 4.7 ppm and 118 ppm are used for the ^1^H and ^15^N dimension, respectively. The total acquisition time for each experiment is 135 min.
  c. Process the spectra using the NMRPipe software.^10^
  d. Analyze the obtained spectra (Figure 8) using the POKY^11^ or NMRFAM SPARKY.^12^

***Alternatives:*** Alternative NMR software for processing include Bruker TopSpin 4.2.0, and earlier releases, the CcpNmr software suite, or software available in NMRBox software collection.^21^^,^^22^ An extensive review of NMR data processing is presented in Stern and Hoch (1996).^23^

Sequence-specific resonance assignments for SARS-CoV-2 3CL^pro^ are available in the BMRB entry 50780^24^ and can be used to validate the spectrum and to label the observed peaks following the nearest neighbor criterium.

### Crystallization and structural determination of SARS-CoV-2 3CL^pro^

**Timing: Approximately 2 weeks**

***Note:*** SARS-CoV-2 3CL^pro^ can be crystallized at 3–8 mg mL^−1^ through vapor diffusion technique (hanging drop method) using a 20–60 mM ammonium acetate buffer at pH 7.0 and 20–30 % PEG4000 as a precipitant. This protocol reports a representative crystallization procedure using a 5 mg mL^−1^ sample.

16. Crystallization of SARS-CoV-2 3CL^pro^.
  a. Prepare the crystallization cocktail by filling each well (0.5 mL) of the EasyXtal 15-well crystallization plate (Quiagen) with the correct volumes of 1 M ammonium acetate at pH 7.0 (AMA 1 M), 50 % PEG4000 (PEG 50 %), and MilliQ water according to the scheme in Table 1, as shown in Figure 9A.
    ***Note:*** Due to different viscosities of the three solutions added, it is recommended to mix the content of each well using a micropipette.

**Table 1:** Preparation of the crystallization plate

[Ammonium acetate at pH 7.0] (mM)						
P E G 4 0 0 0 (%)		20	30	40	50	60
	20	AMA 1 M = 10 μL	AMA 1 M = 15 μL	AMA 1 M = 20 μL	AMA 1 M = 25 μL	AMA 1 M = 30 μL
		PEG 50 % = 200 μL	PEG 50 % = 200 μL	PEG 50 % = 200 μL	PEG 50 % = 200 μL	PEG 50 % = 200 μL
		H_2_O = 290 μL	H_2_O = 285 μL	H_2_O = 280 μL	H_2_O = 275 μL	H_2_O = 270 μL
	25	AMA 1 M = 10 μL	AMA 1 M = 15 μL	AMA 1 M = 20 μL	AMA 1 M = 25 μL	AMA 1 M = 30 μL
		PEG 50 % = 250 μL	PEG 50 % = 250 μL	PEG 50 % = 250 μL	PEG 50 % = 250 μL	PEG 50 % = 250 μL
		H_2_O = 240 μL	H_2_O = 235 μL	H_2_O = 230 μL	H_2_O = 225 μL	H_2_O = 220 μL
	30	AMA 1 M = 10 μL	AMA 1 M = 15 μL	AMA 1 M = 20 μL	AMA 1 M = 25 μL	AMA 1 M = 30 μL
		PEG 50 % = 300 μL	PEG 50 % = 300 μL	PEG 50 % = 300 μL	PEG 50 % = 300 μL	PEG 50 % = 300 μL
		H_2_O = 190 μL	H_2_O = 185 μL	H_2_O = 180 μL	H_2_O = 175 μL	H_2_O = 170 μL

**Table 2:** Data collection, processing and refinement statistics (PDB code 8CDC)

	8CDC
**Data collection and processing**	

Wavelength (Å)	0.9762
Detector	EIGER 16M
Crystal-to-Detector distance (mm)	180.413
Oscillation angle (degrees)	0.1
Number of images	3600
Space group	C121
Unit cell (*a, b, c,* Å)	114.1 53.5 44.6
Resolution range (Å)^a^	55.57 - 1.54 (1.57 - 1.54)
Total number of reflections^a^	254428 (12272)
Unique reflections^a^	38825 (1885)
Multiplicity^a^	6.6 (6.5)
Completeness^a^ (%)	100.0 (100.0)
R_sym_^a^^,^^b^ (%)	4.7 (96.1)
R_pim_^a^^,^^c^^28^ (%)	3.0 (62.1)
Mean I half-set correlation CC(1/2)^a^	0.999 (0.758)
Mean I/σ(I)^a^	15.3 (1.6)

**Refinement statistics**	

Number of monomers in the asymmetric unit	1
R_factor_^d^ (%)	18.5
R_free_^d^ (%)	23.9
Cruickshank’s DPI for coordinate error^e^ based on R_factor_ (Å)	0.097
Wilson plot B-factor (Å^2^)	22.7
Average all atom B-factor (Å^2^)	28.1
RMS (bonds)^d^	0.013
RMS (angles)^d^	1.81
Total number of atoms	2574
Total number of water molecules	294
Solvent content (%)	36.40
Matthews Coefficient (Å^3^/Da)	1.93

**Ramachandran plot**	

Most favored regions (%)	97.0
Additionally allowed regions (%)	2.6
Disallowed regions (%)	0.4

a Highest resolution bin in parentheses;

b $R_{sym}=\sum_{hkl}\sum_{j}\left|I_{j}-\left\langle I\right\rangle \right|/\sum_{hkl}\sum_{j}I_{j}$ , where I is the intensity of a reflection, and ⟨I⟩ is the mean intensity of all symmetry related reflections j;

c $R_{p.i.m.}=\sum_{hkl}\left\{{\left[1/\left(N-1\right)\right]}^{1/2}\sum_{j}\left|I_{j}-\left\langle I\right\rangle \right|\right\}/\sum_{hkl}\sum_{j}I_{j}$, where I is the intensity of a reflection, and ⟨I⟩ is the mean intensity of all symmetry related reflections j, and N is the multiplicity^28272827282828282726252626272727272727272625242322222118^;

d Taken from REFMAC; R_free_ is calculated using 5% of the total reflections that were randomly selected and excluded from refinement;

e DPI=Rfactor·Dmax·compl−13Natoms(Nrefl−Nparams), where *N*_*atoms*_ is the number of the atoms included in the refinement, *N*_*refl*_ is the number of the reflections included in the refinement, *D*_*max*_ is the maximum resolution of reflections included in the refinement, *compl* is the completeness of the observed data, and for isotropic refinement, *N*_*params*_ ≈ *4N*_*atoms*_

**Table 3:** Yields of pure native 3CL^pro^ obtained in different media

Medium	Yield (mg L^−1^)
Super broth	122
LB	82
MJ9 (minimal medium)	28

Protein was spectrophotometrically quantified assuming MW = 33,796 Da and ε_280_ = 32,890 A.U. M^−1^ cm^−1^

**Figure 9:**
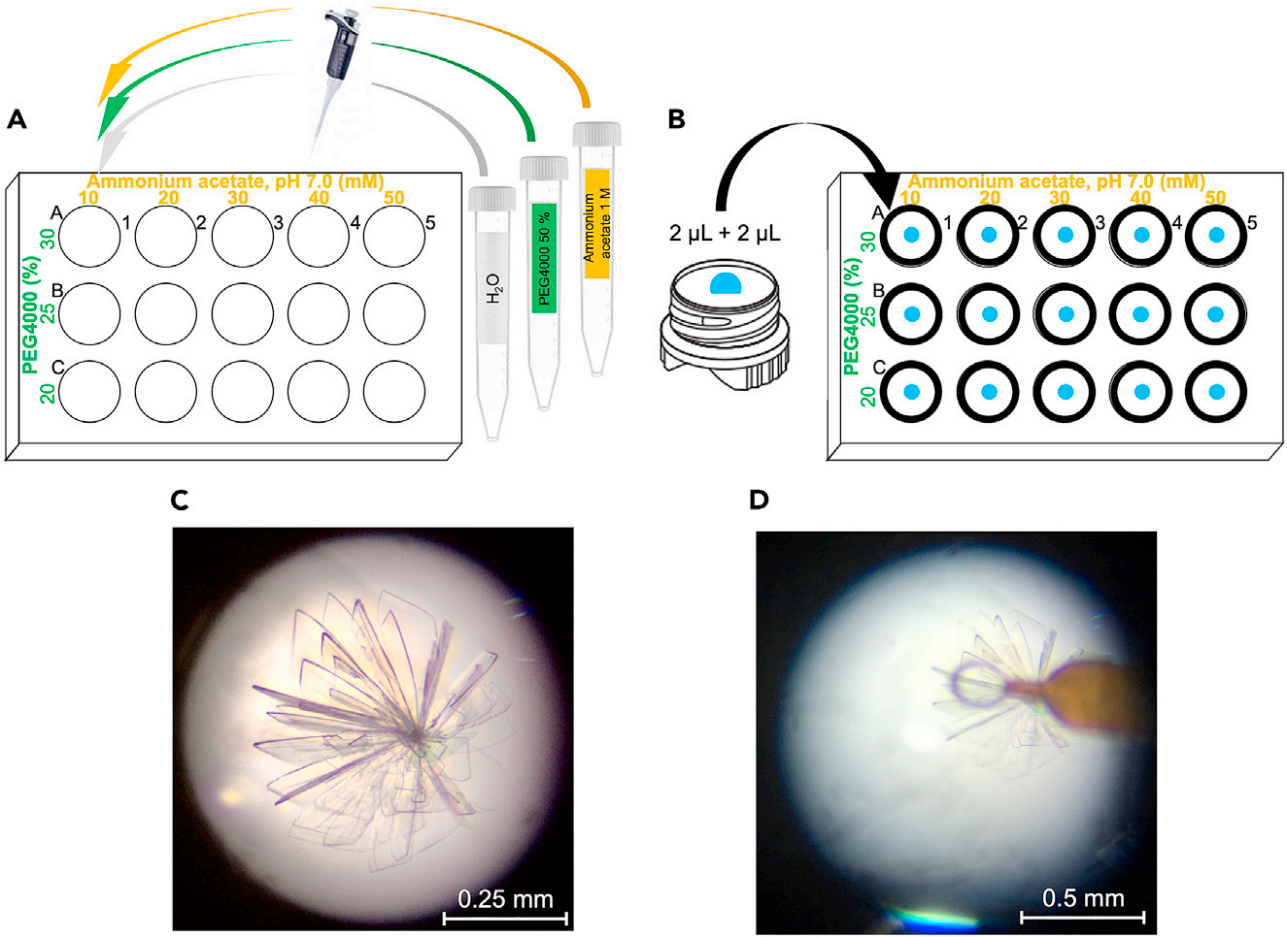
Experimental set up for the crystallization of SARS-CoV-2 3CL^pro^ by hanging drop and crystal recovery and storage (A and B) (A) Preparation of the crystallization plate, and (B) crystallization drops deposition. (C and D) (C) 3CL^pro^ typically grows as bundles of sword-shaped crystals which (D) are fished using cryo-loops, flash-cooled in liquid nitrogen for storage, and shipped to the synchrotron facility for data collection.
  b. Gently thaw 50 μL of purified 5 mg mL^−1^ 3CL^pro^ on ice.
  c. Place a screw-in crystallization support upside down and pipette 2 μL of 3CL^pro^ solution at its center.
  d. Recover 2 μL of the precipitation cocktail present in well A1 and pipette it onto the already deposited protein solution.
  e. Reverse the crystallization support in the original position and screw it onto the corresponding A1 well (Figure 9B).
  f. Repeat point c for all the wells present in the crystallization plate.
  g. Store the crystallization plate at 20 °C in a PKS-E100 Protein Crystallization Cabinet (RUMED) to avoid temperature oscillations as well as vibrations during crystallization.
  h. Monitor the evolution of each crystallization drop under a light microscope after regular time intervals (1 day, 2 days, 4 days, 1 week and then every week), noting any phase transition occurring (precipitation, liquid-liquid separation, crystal formation, or clear drop).
    ***Alternatives:*** The same experimental setup used to crystallize native 3CL^pro^ can be also successfully followed, after minor changes, to crystallize the protein in the presence of potential inhibitors by using either co-crystallization or crystal soaking. In the first case, appropriate amounts of test molecule should be added in the crystallization cocktail (point 16a) prior to drop deposition, in order to obtain a co-crystallization drop containing both the protein and the molecule. To perform the crystal soaking, procedure described at point 17c (see below), the protocol should be modified by exposing a crystal of native 3CL^pro^ in a cryo-protectant droplet that also contains the appropriate amount of ligand. During soaking the ligand may diffuse into the preformed crystal through solvent channels and bind to the protein. Exposure time of the protein to the ligand needs to be optimized to achieve complete binding (usually from a few minutes to 12–16 h).
17. Crystal storage and shipping.
  a. Fill a SPINE Sample Changer Basket (Molecular Dimensions) with ten magnetic Cryo-Vials (Molecular Dimensions) and place it in a foam Dewar topped up with liquid nitrogen.
  b. Select the desired 3CL^pro^ crystal candidate(s) (Figure 9C) to be shipped to the synchrotron facility among those grown in one or more wells by carefully observing the crystallization drops under the light microscope.
  c. Open the screw-in crystallization support, fish the crystal from the crystallization drop using a cryo-loop (Molecular Dimensions) of the correct size mounted on a magnetic Cryo wand (Molecular Dimensions) (Figure 9D), and expose it in a new cryo-protectant drop composed of a slightly higher concentration of the crystallization cocktail.
  d. Fish the crystal from the new drop and quickly flash freeze it by depositing the cryo-loop in the magnetic cryo-vial stored in the basket immerged in liquid nitrogen.
  e. Repeat the same procedure for each crystal to be shipped, load the filled baskets in a dry shipper properly equilibrated with liquid nitrogen and ship the material to the synchrotron.

**CRITICAL:** The liquid contained in the drops tends to evaporate quickly. This phenomenon also causes the disruption of the crystal order, which in turn negatively affects data quality. Crystal fishing and flash-cooling procedures must be therefore carried out as quickly as possible. If additional time is needed between two consecutive crystal fishing steps, place the drop support back onto the corresponding well to minimize drop drying.

**Figure 10:**
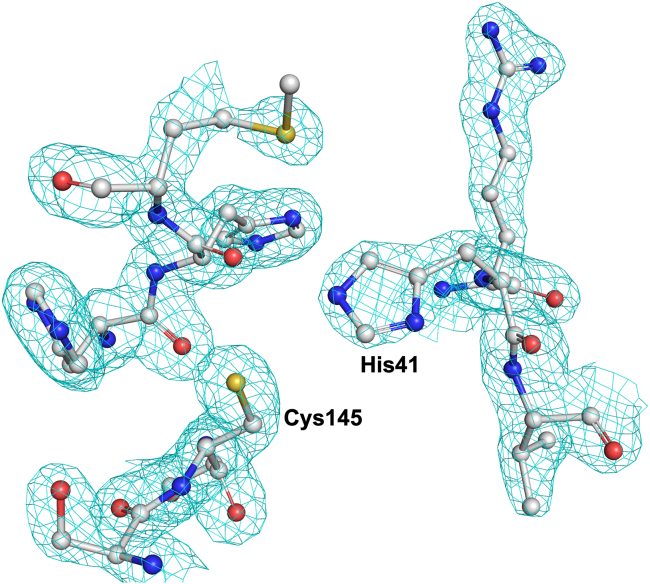
Active site region the X-ray crystal structure of SARS-CoV-2 3CL^pro^ The atomic model is shown superimposed on the final 2Fo - Fc electron density map contoured at 1σ and colored gray. The carbon, nitrogen, oxygen, and sulfur atoms are gray, blue, red, and yellow, respectively. The figure is generated using PyMol (The PyMOL Molecular Graphics System, v. 1.8 Schrödinger, LLC.).

18. Crystallography data collection, processing, and structural determination.
  ***Note:*** Diffraction data used for the structural determination of the X-ray crystal structure of native SARS-CoV-2 3CL^pro^ were collected on one crystal at 100 K using synchrotron X-ray radiation at the EMBL P13 beamline of the Petra III storage ring, c/o DESY, Hamburg (Germany).^25^
  a. Record diffraction data using the collection strategy suggested by the MXCuBE software.^13^^,^^14^
  b. Process X-ray diffraction images by using XDS.^15^
  c. Scale and reduce processed data by using AIMLESS,^17^ included in the suite CCP4.^16^
  d. Carry out structural refinement and model building using the REFMAC5 software^18^ and COOT,^26^ respectively, both included in the suite CCP4.^16^ Use the structure factor amplitudes obtained by AIMLESS as experimental data and the PDB entry 7ALH as a starting model until refinement convergence. A representative picture of the final X-ray crystal structure of 3CL^pro^ obtained in this work (and deposited in the Protein Data Bank under PDB code 8CDC) is shown in Figure 10. Data collection, processing and refinement statistics are reported in Table 2.
    ***Alternatives:*** Other data collection facilities may be used. Other software can be used for analyzing the crystallographic data, such as PHENIX.^27^
    ***Note:*** Crystals grown in experimental conditions described belong to space group C121, as does the starting model selected for the refinement (PDB code 7ALH). If the space groups of experimental data and the starting model do not match, a molecular replacement procedure must be carried out to obtain initial crystallographic phases to be used in the following refinement and model building steps.

### Sample quality control

In addition to crystallography and NMR data, if available, sample quality control includes SDS-PAGE, MALDI-TOF mass spectrometry, and specific enzyme activity measurements. The expected specific activity is 55 Units / μmole of enzyme, where a Unit is 1.0 μmol substrate cleaved / min, assayed using the FRET buffer conditions, as described in “materials and equipment” section, using 0.4 μM 3CL^pro^, 200 μM DABCYL-KTSAVLQ/SGFRKME-EDANS substrate at 25°C.

## Expected outcomes

This paper provides a detailed protocol for the expression, purification, and production of tens- of- milligram quantities of highly pure (> 99 %) SARS-CoV-2 3CL^pro^ with native N and C termini and in a biochemically-active form, as demonstrated by the bio-structural characterization and activity assays reported. Yields of pure SARS CoV-2 3CL^pro^ obtained, as high at ∼ 120 mg / mL, depend on the medium used for fermentation (Table 3).

3CL^pro^ samples produced by this protocol are stable in NMR measurements for up to 1 week at room temperature and provide excellent HSQC-TROSY spectra suitable for structure, dynamic, ligand binding studies. Moreover, the set-up crystallization protocol provides high quality 3CL^pro^ X-ray crystal structures at resolution better than 2 Å, thus suitable for biophysical and drug discovery research using X-ray crystallography. In the paper a standardized assay for estimating IC_50_ for new potential 3CL^pro^ inhibitors is also presented, thus complementing structural information with biochemical insights of catalytic and inhibition mechanism of this target.

## Limitations

A percentage of the obtained 3CL^pro^ crystals (∼ 20 %) do not diffract at high resolution. The protocol section that describes the characterization of enzyme inhibition by FRET assays assumes solubility of the screening molecules in 4 % DMSO. For potent inhibitors which can be studied at lower concentrations, lower DMSO concentrations may be used if solubility allows. Each potential inhibitor should be evaluated for solubility prior to setting up the assay. Moreover, IC_50_ obtained from the biochemical assays is a preliminary measurement of inhibitor potency. A more accurate investigation of the inhibition should be provided by determining thermodynamic and kinetic parameters for the inhibition mechanism.

## Troubleshooting

### Problem 1

Following the 12–16 h cell growth on LB Petri dishes, no colonies are visible.

### Potential solution

After checking each step of the transformation procedure was carried out correctly (*e.g.*, correct antibiotic, plasmid concentration, duration of heat shock), the concentration of the cell suspension can be increased via centrifugation. At the end of section 13f in the “before you begin” section, centrifuge the cell suspension for 1 min at 2500 × *g* and decant half of the supernatant. Resuspend the pellet in the remaining supernatant for a more concentrated cell culture.

### Problem 2

During cell lysis (step 2. Cell lysis and soluble extract recovery), heat generated by sonication and French Press, can cause aggregation or misfolding of 3CL^pro^

### Potential solution

Sonication should be done in short blasts, with the sample cooled in an ice bath in a container with good thermal conductivity. Lysis through French press should be also performed constantly maintaining the sample in an ice bath. Microfluidizer or homogenizer can be used in place of the aforementioned techniques, though lysis by these methods may be less complete resulting in lower final yields.

### Problem 3

The expressed His_6_-SUMO-3CL^pro^ undergoes a first step of purification through an immobilized nickel affinity chromatography (IMAC) (step 3). Due to the high amount of protein expressed from 1 L of cell culture and a column capacity of usually 10 mg mL^−1^ of resin (indicating that a 5 mL column will efficiently bind up to 50 mg of protein), column overloading issues can be encountered. Such an event is clearly detectable by running an SDS-PAGE of the flow-through, which would show large amounts of unbound His_6_-SUMO-3CL^pro^.

### Potential solution

It is strongly recommended to split the soluble lysate in two or more aliquots and perform the IMAC in successive steps. As an alternative, two or more columns placed in series can also be used to achieve high amounts of protein recovery and speed up the purification procedure.

### Problem 4

Uncontrolled cleavage of His_6_-SUMO tag from His_6_-SUMO-3CL^pro^ probably by trace amounts of native *E. coli* proteases has been observed at the end of step 3 (IMAC), just before the addition of His_8_-MBP-SUMO^pro^.

### Potential solution

Uncontrolled cleavage can be minimized by carrying out cleavage in imidazole immediately after purifying the His_6_-SUMO-3CL^pro^, with low to no negative effects on the production of pure and native 3CL^pro^, or by on-column cleavage, as suggested by this protocol.

### Problem 5

The on-column cleavage protocol set up to perform IMAC purification, SUMO protease cleavage of His_6_-SUMO tag from 3CL^pro^, and removal of cleaved His_6_-SUMO tag and His_8_-MBP-SUMO^pro^ from native 3CL^pro^ in one step is more efficient and quicker. However, in some cases subsequent elution of the proteins reveals incomplete cleavage of the His_6_-SUMO-3CL^pro^, resulting in reduced final yields.

### Potential solution

His_6_-SUMO-3CL^pro^ fusion that elutes from the column can be dialyzed to remove imidazole and undergo a second on-column cleavage procedure.

### Problem 6

Incomplete cleavage of the His_6_-SUMO-3CL^pro^ fusion is observed (*i.e.*, full target fusion is visible in the final elution from the IMAC).

### Potential solution

Collect the elution fractions containing the His_6_-SUMO-3CL^pro^ fusion, perform an 12–16 h dialysis/buffer exchange and simultaneous cleavage in the presence of H_8_-MBP-SUMO^pro^ and repeat an IMAC step by collecting the flow-through.

### Problem 7

No increasing fluorescence signal is observed after combining enzyme and substrate during the FRET Assay control (described in step 12).

### Potential solution

3CL^pro^ is very sensitive to oxidation and can lose enzymatic activity due to oxidation of the active site cysteine even though reducing agents are present. During purification it is advantageous to work quickly and at 4°C as much as possible to minimize this effect. Samples can also be protected from O_2_ by storing them under nitrogen or argon gas.

### Problem 8

Quality of ^15^N HSQC spectrum is not optimal.

### Potential solution

Prepare a ^2^H,^15^N -enriched sample to obtain a higher quality ^15^N HSQC spectrum.

### Problem 9

Reservoir solutions for crystallization trials includes mixing of ammonium acetate, 50 % PEG4000, and water, which have different viscosity and may prevent the final solution to be homogeneous.

### Potential solution

Carefully mix the reservoir solution present in each well using a micropipette.

## Resource availability

### Lead contact

Further information and requests for resources and reagents should be directed to and will be fulfilled by the lead contact, G.T. Montelione, monteg3@rpi.edu.

### Materials availability

Plasmids generated in this study have been deposited to AddGene, and are available under the Uniform Biological Material Transfer Agreement (“UBMTA”):

Plasmid pGTM_CoV2_NSP5_004_SUMO. AddGene ID: 190062.

Plasmid pGTM_CoV2_NSP5_C145A_001_SUMO. AddGene ID: 192349.

Plasmid pGTM_YR375_SUMO_Protease_001. AddGene ID: 190063.

Plasmid sequencing data is available at: https://github.rpi.edu/RPIBioinformatics/SARS-CoV-2-3CLpro.

## Data Availability

Datasets generated during this study, including the NMR free-induction decay (FID), and crystallographic processed and reduced reflections, are available at: Github: RPI Bioinformatics SARS-CoV-2-3CL^pro^ (https://github.rpi.edu/RPIBioinformatics/SARS-CoV-2-3CLpro) The Protein Data Bank accession number for the crystal structure of CoV-2-3CL^pro^ described here is 8CDC
